# Transcriptomic Characterization of a Chicken Embryo Model Infected With Duck Hepatitis A Virus Type 1

**DOI:** 10.3389/fimmu.2018.01845

**Published:** 2018-08-24

**Authors:** Jinyan Xie, Qiurui Zeng, Mingshu Wang, Xumin Ou, Yunchao Ma, Anchun Cheng, Xin-Xin Zhao, Mafeng Liu, Dekang Zhu, Shun Chen, Renyong Jia, Qiao Yang, Ying Wu, Shaqiu Zhang, Yunya Liu, Yanling Yu, Ling Zhang, Xiaoyue Chen

**Affiliations:** ^1^Institute of Preventive Veterinary Medicine, Sichuan Agricultural University, Chengdu City, China; ^2^Key Laboratory of Animal Disease and Human Health of Sichuan Province, Sichuan Agricultural University, Chengdu City, China; ^3^School of Medicine, Shanghai Jiao Tong University, Shanghai, China; ^4^Avian Disease Research Center, College of Veterinary Medicine, Sichuan Agricultural University, Chengdu City, China

**Keywords:** Duck hepatitis A virus type 1, transcriptomic analysis, chicken embryo models, innate immune system, *SOCS*

## Abstract

Duck hepatitis A virus type 1 (DHAV-1) is one of the most common and lethal pathogens in young ducklings. Live-attenuated DHAV vaccine (CH60 strain) developed by passaging in chicken embryos provided effective immune protection for ducklings. However, the accurate mechanism for such adaption in chicken embryos is not fully revealed. Here, we utilize RNA-sequencing to perform global transcriptional analysis of DHAV-1-innoculated embryonated livers along with histopathological and ultrastructural analysis. This study revealed that infection with DHAV-1 strain CH60 is associated with enhanced type I and II interferon responses, activated innate immune responses, elevated levels of suppressor of cytokine signaling 1 and 3 (*SOCS1* and *SOCS3*) accompanied with abnormalities in multiple metabolic pathways. Excessive inflammatory and innate immune responses induced by the CH60 strain are related to severe liver damage. Our study presents a comprehensive characterization of the transcriptome of chicken embryos infected with DHAV-CH60 and provides insight for in-depth exploration of viral adaption and virus–host interactions.

## Introduction

Duck hepatitis A virus type 1 (DHAV-1), a member of the genus *Avihepatovirus* with characteristics of the family *Picornaviridae* (1–8), was first reported in Long Island, New York in 1945. DHAV-1 poses a serious threat to the duck industry worldwide (9, 10). There was recently an outbreak of DHAV-1 infection in Japan, and the nucleotide sequences of outbreak PCR products exhibited 96% identity with the HB02 strain of DHAV-1, which was isolated in China (11). Therefore, this virus may have been introduced from an epidemic area into a pest-free area. DHAV-1 is one of the most common and lethal pathogens in young ducklings and is responsible for acute hepatitis characterized by petechial and ecchymotic hemorrhages of liver surfaces (12–14). Due to the need for prevention and control of this disease, many studies have focused on diagnostic methods (15–20).

Current studies on the pathogenicity of DHAV-1 have been limited to the innate immune response at the transcription level (21–24). However, the complex immunological mechanisms associated with DHAV-1 remain ill-defined, which is due to the lack of an available chicken embryo model. Previous studies have proposed DHAV-1 infection in mature ducks as an alternative small-animal model for viral hepatitis (21). Nonetheless, there are limitations associated with duck models, such as the lack of duck-derived antibodies and the lack of stable animal sources.

The chicken embryo model is commonly used to investigate vertebrate biology (25), and several studies have utilized this animal model to explore complex mechanisms in recent years (26–29). Our lab has previously cultivated a chicken embryo-attenuated strain of DHAV-1 that causes liver disease in ducks in a manner similar to DHAV-1 (30). Herein, we describe the first global transcriptional study of the liver in a specific-pathogen-free (SPF) chicken embryo model of DHAV-1 infection. The study was undertaken to explore virus–host interactions in detail and provide insight into an alternative small-animal model.

## Materials and Methods

### Ethics Statement

The study was approved by the Committee of Experiment Operational Guidelines and Animal Welfare of Sichuan Agricultural University (the approved permit number is XF2014-18). Experiments were conducted in accordance with approved guidelines.

### Viruses and Animals

The virulent DHAV-1 CH strain and the attenuated DHAV-1 CH60 strain were provided by the Institute of Preventive Veterinary Medicine, Sichuan Agricultural University. SPF embryonated eggs were infected with the CH strain at a concentration of 10^7.88^ copies/ml and the CH60 strain at a concentration of 10^7.65^ copies/ml *via* the allantoic cavity. Viral copies were determined by quantitative reverse transcription-polymerase chain reaction (qRT-PCR) (16).

Specific-pathogen-free embryonated eggs were purchased from Beijing Merial Vital Laboratory Animal Technology Co., Ltd. All eggs were incubated at 37.8°C in the same incubator and candled daily.

### Experimental Procedures

Twelve-day-old embryonated eggs were randomly divided into three groups (15 embryonated eggs in each group). The embryonated eggs in the first group (CH group) received 0.12 ml of the DHAV-CH strain (10^7.88^ copies/ml) *via* allantoic cavity inoculation, the embryonated eggs in the second group (CH60 group) received 0.20 ml of the DHAV-CH60 strain (10^7.65^ copies/ml) *via* allantoic cavity inoculation, and the final group (mock group) was inoculated with 0.20 ml of 0.75% physiological normal saline as a negative control. Three embryonated egg livers were collected from each group at 12, 24, and 36 h post infection (hpi), and six embryonated eggs livers were collected from each group at 48 hpi. The right lobes of the liver specimens were immediately cryopreserved in liquid nitrogen until RNA isolation was performed. Additionally, the left lobes of the liver specimens were split into two components: one was soaked in 4% paraformaldehyde solution for histopathological examination, and the other was soaked in 2.5% glutaraldehyde solution for electron microscopy.

### Hematoxylin and Eosin (HE) Staining, TUNEL Assay, and Electron Microscopy

The livers soaked in 4% paraformaldehyde solution were dehydrated, embedded in paraffin, cut into 4-μm-thick sections, and stained with HE using standard procedures (31).

Four-micron sections from the CH60, CH and mock group at 48 hpi were also used to perform terminal dUTP nick end labeling (TUNEL) using an *In Situ* Apoptosis Detection Kit (Boster Inc., Wuhan, China) according to the manufacturer’s instructions. Apoptotic cells were observed under a light microscope. The apoptotic nuclei were counted in four randomly selected non-overlapping fields.

The livers from the CH60, CH, and mock groups obtained at 48 hpi were post-fixed in 1.0% osmium tetroxide. After a stepwise dehydration in acetone, samples were embedded in epoxy resin 618 and polymerized at 80°C for 72 h. Then, 50-nm ultra-thin sections were prepared, collected on grids, and stained with uranyl acetate and lead citrate for subsequent examination with a Tecnai G2 F20 transmission electron microscope.

### RNA Isolation, cDNA Library Construction, and RNA Sequencing

Total RNA from the livers collected at 48 hpi was extracted using a mirVana miRNA Isolation Kit (Ambion) following the manufacturer’s protocol. There were three biological replicates for each group, with each replicate comprising pooled RNA from two embryonated eggs. RNA integrity was evaluated using an Agilent 2100 bioanalyzer (Agilent Technologies, Santa Clara, CA, USA). Samples with RNA integrity numbers (RINs) > 7 were subjected to subsequent analysis. Libraries were constructed using a TruSeq Stranded mRNA LT Sample Prep Kit (Illumina, San Diego, CA, USA) according to the manufacturer’s instructions. Then, these libraries were sequenced on an Illumina sequencing platform (HiSeqTM 2500 or Illumina HiSeq X 10), and 125/150-bp paired-end reads were generated.

### Quantitative RT-PCR

Gene expression levels were determined by performing qPCR using a SYBR^®^ Premix Ex Taq™ II (Tli RNaseH Plus) Kit (TaKaRa) and an Applied CFX96 real-time PCR detection system (Bio-Rad, Hercules, CA, USA). Primer sequences were specifically designed for suppressor of cytokine signaling 1 (*SOCS-1*), *SOCS-3, STAT1, STAT3, IRF-1, IRF-7, IFN-*α, *IFN-*β, *IFN-*γ, *IL-6, IL-10, TLR3, TLR7*, and *GAPDH* using Primer Premier 5 (Table S1 in Supplementary Material). Amplification was performed in 10-µl reactions containing 0.4 µl of each primer and 1 µl of cDNA. The following thermal cycling conditions were used: initial activation at 95°C for 30 s, 40 cycles of denaturation at 95°C for 5 s, and annealing and extension at 58.6°C for 30 s, and a dissociation curve analysis step. Fold change was determined using the 2^−ΔΔCt^ method (32).

### Analysis of Differentially Expressed Genes (DEGs), Cluster Analysis, and Gene Ontology (GO) and KEGG Enrichment

FPKM (33) and read count values of each transcript (protein coding) were calculated using Bowtie 2 (34) and eXpress (35). DEGs were identified using the DESeq (36) (2012) functions estimateSizeFactors and nbinomTest. *P*-value <0.05 and fold change >2 or fold change <0.5 were set as the thresholds for significant differential expression. Hierarchical cluster analysis of the DEGs was performed to explore the transcript expression pattern. GO enrichment and KEGG (37) pathway enrichment analysis of the DEGs were performed using the hypergeometric distribution test with the Phyper function in the R software package. Significantly-enriched unigenes were selected based on a *q*-value of 0.05 (adjusted *p*-values were found using an optimized FDR). The distribution of unigenes within each GO/pathway category was determined by mapping all differentially expressed unigenes to terms in the GO and KEGG databases.

### Methylation-Specific PCR (MS-PCR)

*SOCS3* gene methylation status in the livers of the mock, CH, and CH60 groups was analyzed by MS-PCR with DNA treated with an EZ DNA Methylation-Lightning™ Kit (Zymo Research, Orange, CA, USA) according to the manufacturer’s instructions. The primers for the methylated sequences were FM-*SOCS3* (5′-TGTTAACGGGTATTTGGATTTTTAC-3′) and RM-*SOCS3* (5′-CCTAACACTCCTCTACTTACACGAA-3′), and the primers for the unmethylated sequences were FU-*SOCS3* (5′-TTAATGGGTATTTGGATTTTTATGA-3′) and RU-*SOCS3* (5′-TCCCTAACACTCCTCTACTTACACAA-3′). PCR was performed in a total volume of 10 µl, containing 5 µl of 2 × TSINGKE Master Mix, 0.3 µl of each primer, and 0.5 µl of treated DNA. Cycling conditions were as follows: the reaction was hot-started at 95°C for 5 min, followed by 32 cycles of denaturation at 95°C for 60 s, annealing at 55°C for 30 s, and extension at 72°C for 30 s; then, a final extension was performed at 72°C for 7 min. Ten microliters of the PCR product was electrophoresed on a 1.5% agarose gel stained with ethidium bromide and imaged under UV light. Normal liver tissue DNA treated with SssI methylase and modified with sodium bisulfite according to the manufacturer’s instructions (New England Biolabs, Beverly, MA, USA) was used as a positive control, and ddH_2_O was used as a negative control.

## Results

### Gross Lesions and Histopathological Analysis

**Figure 1:**
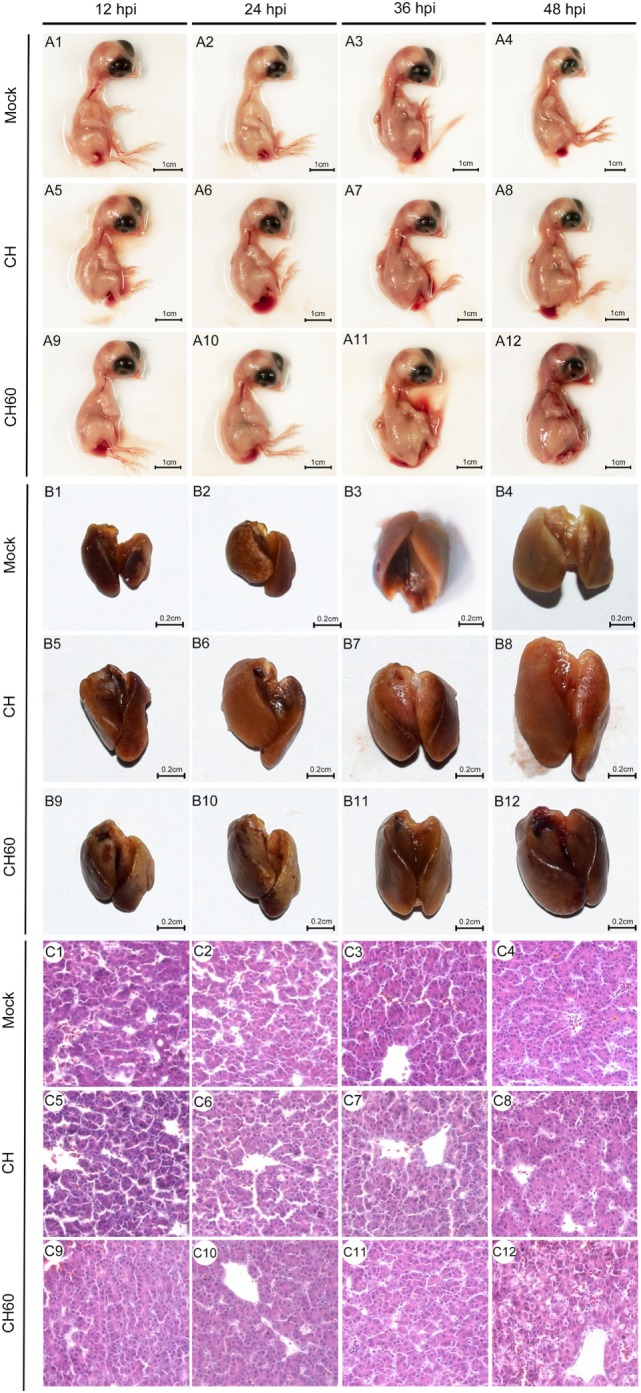
Gross lesions and histopathological lesions in CH- or CH60-infected livers of embryonated eggs. Embryonated eggs were infected with the CH or CH60 strain, and then the livers were collected and soaked in 4% paraformaldehyde solution at 12, 24, 36, and 48 hpi. Microscopic lesions were observed under an optical microscope; magnification, 600×.

Embryonated eggs were collected at 12, 24, 36, and 48 hpi, and we found that the gross lesions of the CH60-infected embryos underwent dynamic changes and peaked at 48 hpi, exhibiting obvious hemorrhage and swelling (Figure 1-A12). However, none of the CH-infected embryonated eggs exhibited gross lesions. Then, liver sections were collected and soaked in 4% paraformaldehyde solution for histopathological examination. We observed ecchymotic hemorrhage in the livers of CH60-infected embryonated eggs at 48 hpi (Figure 1-B12), which corresponded to histopathological lesions. We observed infiltration of the hepatic sinusoids by large numbers of red blood cells, which was accompanied by disordered hepatic cords in the livers of CH60-infected embryonated eggs at 48 hpi. Additionally, parts of the cell nuclei underwent pyknosis, karyolysis, or karyorrhexis (Figure 1-C12). For further histopathological analysis, we used electron microscopy to observe the livers of CH60-infected embryonated eggs at 48 hpi.

### Electron Microscopy and Apoptotic Analysis

**Figure 2:**
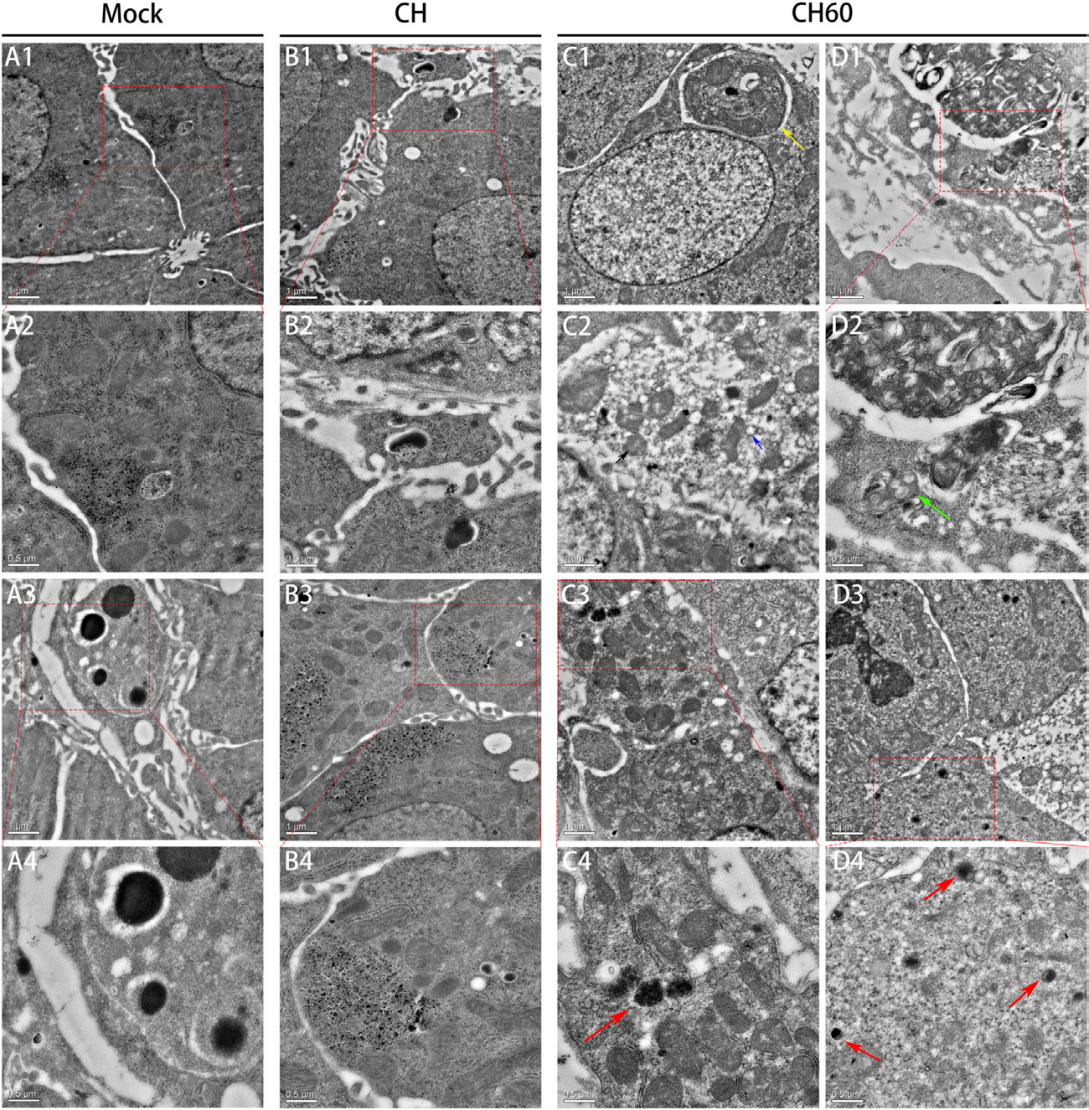
Transmission electron microscopy analysis of liver tissue of the mock, CH and CH60 groups at 48 hpi. A: mock group; A2 and A4 are magnifications of the red boxes in A1 and A3, respectively. B: CH group. B2 and B4 are magnifications of the red boxes in B1 and B3, respectively. C and D: CH60 group. C4, D2, and D4 are magnifications of the red boxes in C3, D1, and D3, respectively.

Transmission electron microscopy (TEM) analysis of the embryonated egg liver tissue samples from the mock, CH, and CH60 groups at 48 hpi is shown in Figure 2. TEM images of the mock group showed clear nuclear structures and mitochondria in the hepatocytes (Figure 2A1,A2), and hepatic sinus endothelial cells were detected in the mock group (Figure 2A3,A4). There were no obvious differences in the nuclear and mitochondrial structures of the mock and CH groups (Figure 2B1); however, hepatic lipoid drops and autophagic vesicles were detected in the hepatocytes of the CH group (Figure 2B4). Compared to the mock and CH60 groups, hepatocytes in the CH group showed the accumulation of large amounts of glycogen (Figure 2B3,B4). TEM images of the CH60 group exhibited obvious pathologic changes, including the exit of large numbers of lipoid drops from the hepatocytes, expansion of the rough endoplasmic reticulum (Figure 2C2, blue arrow), and swelling of the mitochondria where the ridge was fractured (Figure 2C2, black arrow). Moreover, there were abundant secondary lysosomes (Figure 2C3,C4,D3,D4, red arrow) and lysosomal residues (Figure 2D2, green arrow), and organelles such as mitochondria were autophagocytosed (Figure 2C1, yellow arrow).

**Figure 3:**
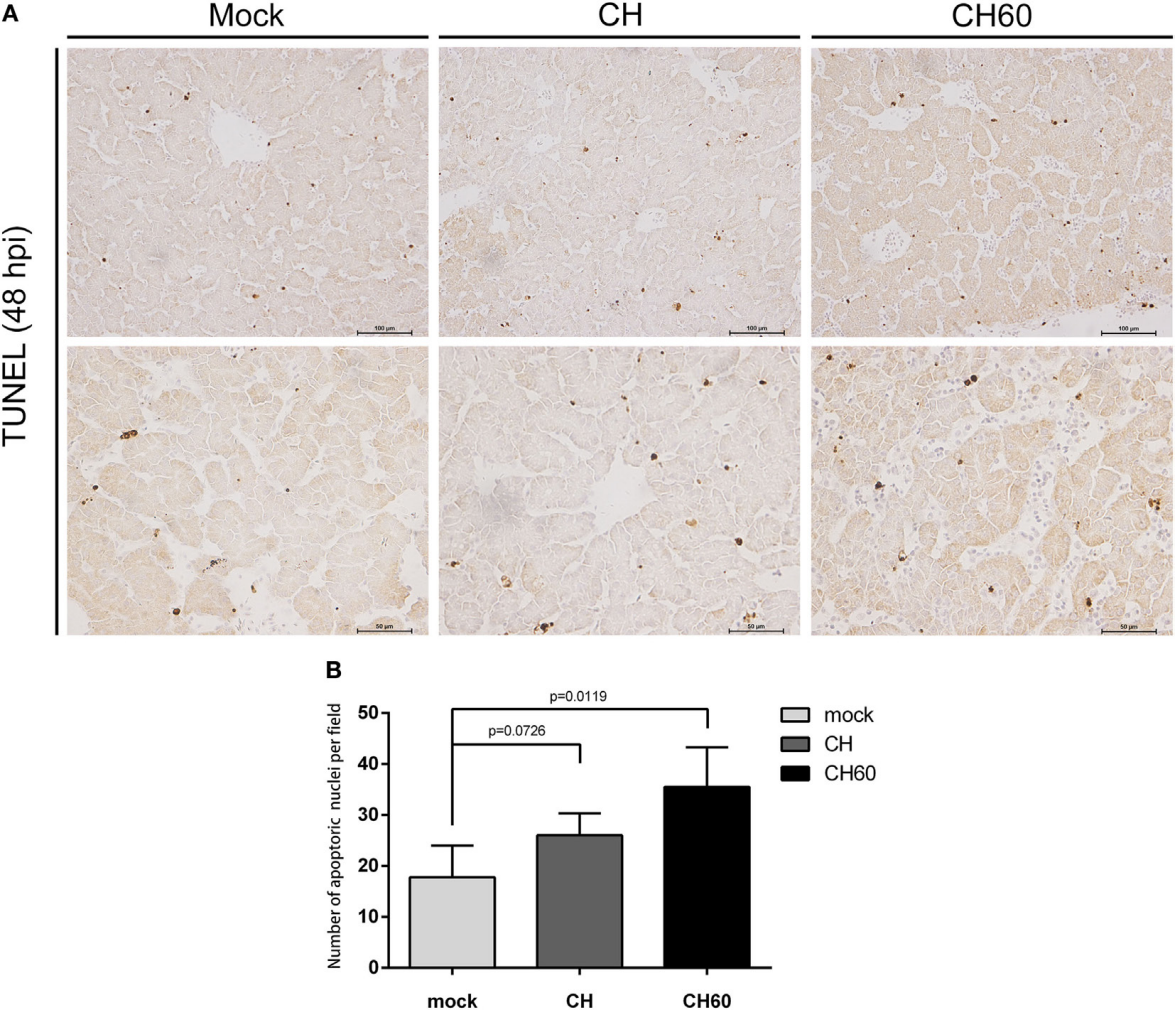
TUNEL assay of the livers of CH- or CH60-inoculated embryonated eggs at 48 hpi. **(A)** TUNEL staining of the livers of the mock, CH, and CH60 groups at 48 hpi. **(B)** Number of apoptotic nuclei identified by TUNEL staining. Student’s *t-*test was used for statistical analysis to compare the mock and treated groups.

Then, we also stained the same liver tissue to detect TUNEL and counted the number of apoptotic nuclei. We observed obvious differences in apoptosis between the CH60 and mock groups (*p* = 0.011, Figure 3B); interestingly, most of the apoptotic cells were detected in the hepatic sinusoids (Figure 3A).

### Transcriptional Profiling of SPF Embryonated Eggs Infected With the DHAV-1 CH or CH60 Strain

We performed transcriptional profiling of CH60-infected livers using the most severe lesions. Total RNA from the livers (48 hpi) of CH- or CH60-infected embryonated eggs and from the livers of mock-infected embryonated eggs was analyzed by RNA-Seq. Three biological replicates, each comprising pooled RNA from two embryonated eggs, were sequenced using three lanes of the Illumina HiSeq 2000 platform. Quality control analyses and read alignment data are shown in Figure S2 in Supplementary Material. DESeq Software was used to identify DEGs in the different infected livers. The list of DEGs (*q* < 0.01 and fold change > 4) and the up- and downregulated genes are provided in Table S2 in Supplementary Material.

### Differential Gene Expression Analysis

**Figure 4:**
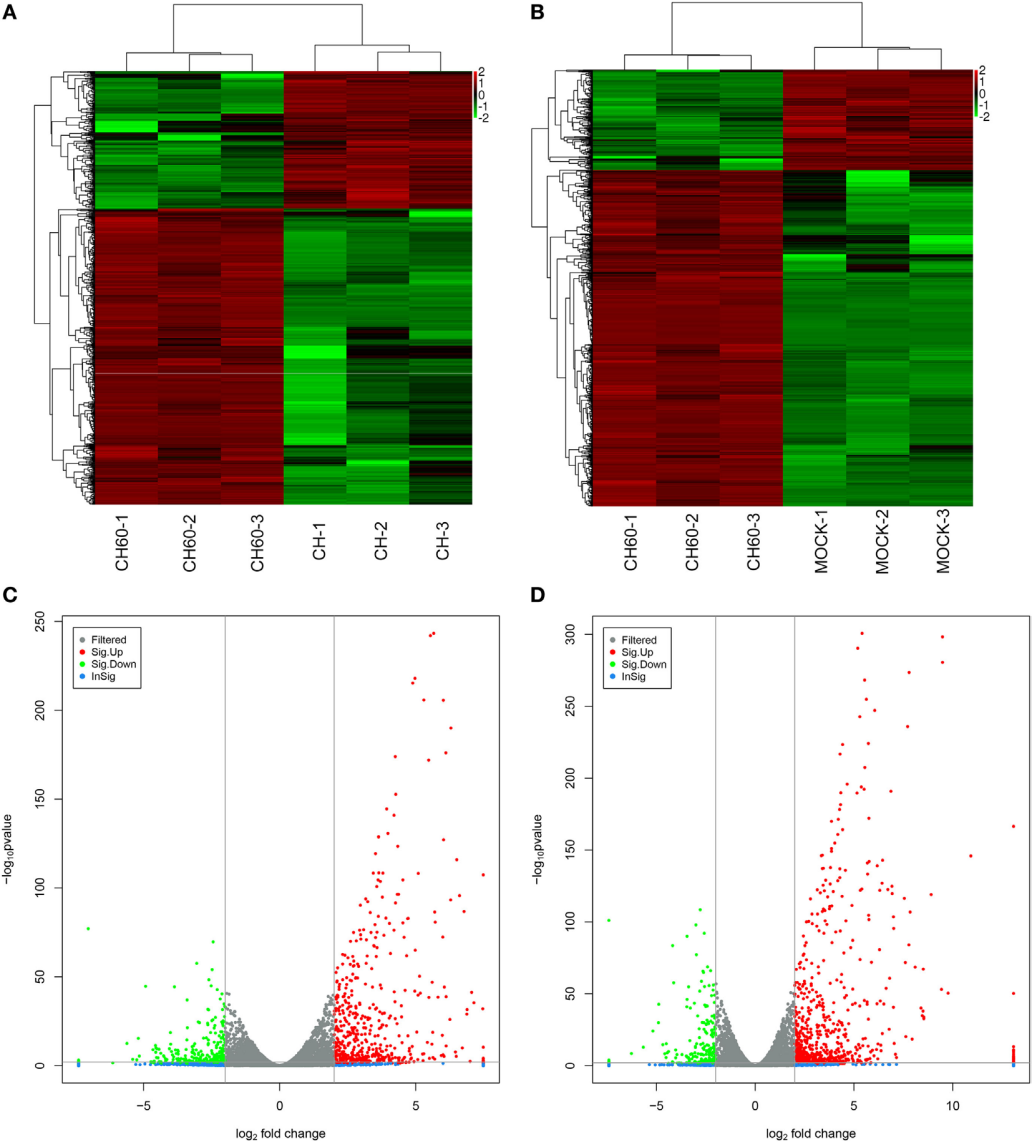
Analysis of differentially expressed genes (DEGs) of the mock, CH, and CH60 groups (*p*-value < 0.01 and fold change > 4). A heatmap was used to classify the gene expression patterns, and a volcano plot displayed the number of DEGs. **(A,B)** The *x*-axis represents the experimental conditions. **(C)** Volcano plot of DEGs between the CH and CH60 groups. **(D)** Volcano plot of DEGs between the MOCK and CH60 groups. The *y*-axis indicates the negative logarithm of the *p*-value; the *x*-axis indicates the base 2 logarithm of fold change.

We analyzed the DEGs among the mock, CH, and CH60 groups with DESeq2. Compared with the mock group, the CH and CH60 groups contained 150 and 2,336 DEGs, respectively (*p*-value < 0.05 and fold change > 2). In addition, there were 2,191 DEGs between the CH60 and CH groups, 1,732 of which were shared between the CH60 and mock groups (Table 1). To further analyze the DEGs, we selected more stringent filter conditions (*p*-value < 0.01 and fold change > 4). Following comparison with the mock and CH groups, there were 844 and 718 DEGs in the CH60 group, respectively. These results were clearly visualized by clustering the samples by differential treatment and by constructing a volcano plot of the DEGs (Figure 4).

**Table 1:** Number of differentially expressed genes between comparisons*.

Individual comparisons			Overlapping genes between comparisons		
	
Comparison	*p*-Value < 0.05 FC > 2	*p*-Value < 0.01FC > 4	Mock vs CH	Mock vs CH60	CH vs CH60
Mock vs CH	150	10	–	72	73
Mock vs CH60	2,336	844	24	–	1,732
CH vs CH60	2,191	718	20	603	–

*Number above “–” indicates DEG at p-value < 0.05 and fold change > 2 between indicated comparisons. Number below “–” indicates DEG at p-value < 0.01 and fold change > 4 between indicated comparisons.

### DHAV-1-Mediated Changes in Expression

There was a clear difference in the number of DEGs between the CH and CH60 groups compared with the mock group (the CH group had 150 DEGs, and the CH60 group had 2,336 DEGs; *p*-value < 0.05 and fold change > 2). These DEGs reflected the differential responses of the host to these two different virulent viruses. Therefore, we performed GO analysis and KEGG pathway analysis to filter the biological processes and pathways impacted by DHAV-1. When comparing the CH group to the mock group, 150 genes were observed to be significantly differentially expressed (*p*-value < 0.05 and fold change > 2), and most of these genes are involved in interactions between neuroactive ligands and receptors, such as *CHRNA4, GABRB2, GRIA4, GRM7*, and *OPRM1* (Figure S4B in Supplementary Material). However, comparison of the CH60 and mock groups yielded 844 DEGs (*p*-value < 0.01 and fold change > 4) involved in multiple biological processes and pathways.

**Figure 5:**
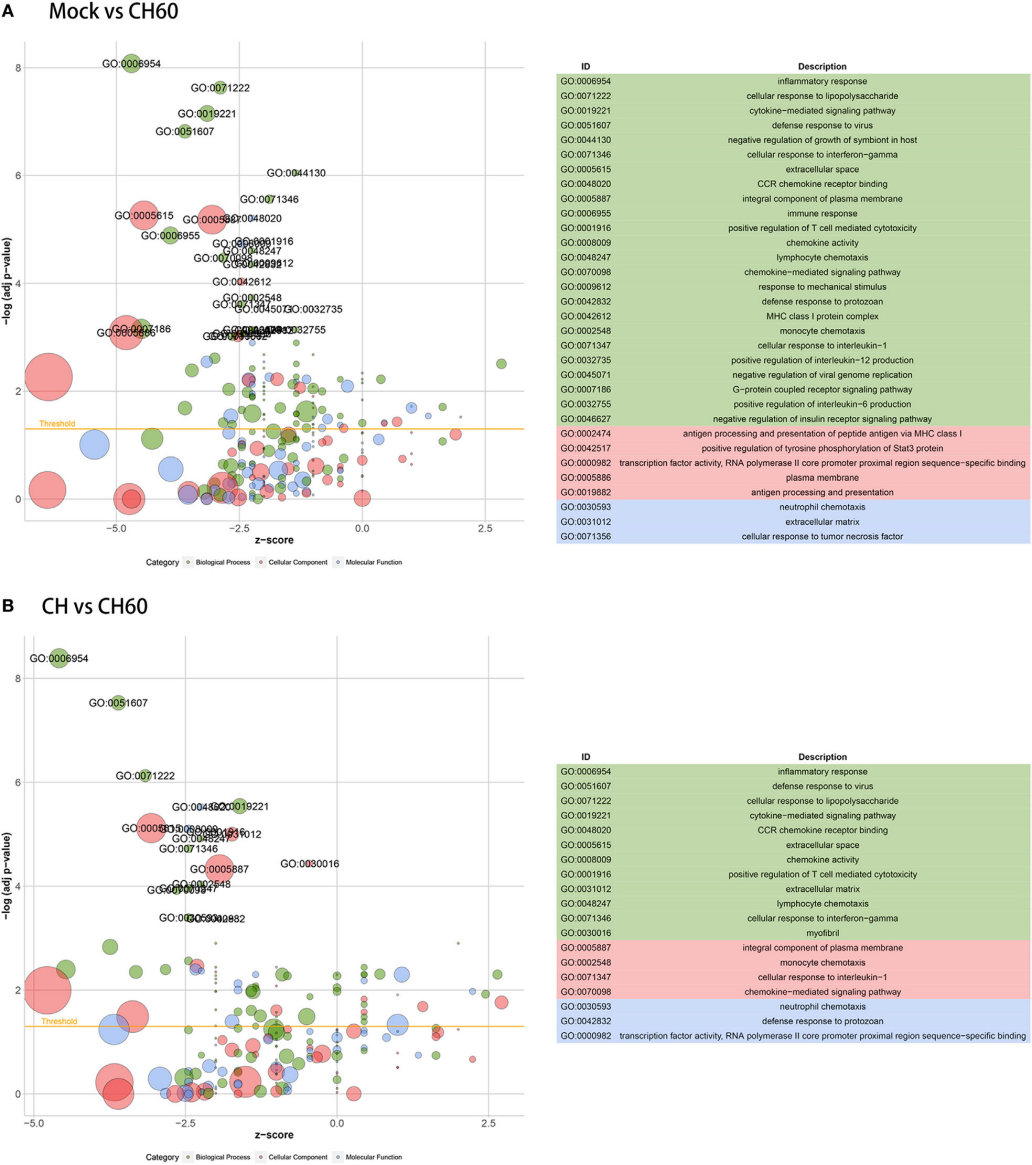
Gene ontology (GO) enrichment analysis of differentially expressed genes (DEGs). The *z*-score is assigned to the *x*-axis, and the negative logarithm of the adjusted *p*-value is assigned to the *y*-axis. The areas of the displayed circles are proportional to the number of genes assigned to the term, and the colors correspond to the categories. **(A)** GO enrichment analysis of DEGs between the mock group and CH60 group (*p*-value < 0.01 and fold change > 4). **(B)** GO enrichment analysis of DEGs between the CH group and CH60 group (*p*-value < 0.01 and fold change > 4).

Gene ontology enrichment indicated that multiple vital biological processes are involved in CH60 strain infection (Figure 5A), including inflammatory response, cytokine-mediated signaling pathway, defense response to virus, cellular response to interferon gamma, and immune response. By tracking the genes involved in these biological processes, we found *IL10* (FC = 371.6) and *IL18* (FC = 8.8) to be significantly upregulated in the inflammatory response. *SOCS1* (FC = 45.8), *SOCS3* (FC = 117.6), and *IFN-*β (FC = INF) participate in the cytokine-mediated signaling pathway; and *IL6* (FC = INF), *OASL* (FC = 1,526.6), *IFIT5* (FC = 1,558.6), *TLR3* (FC = 11.3), and *IRF1* (FC = 46.9) are involved in the defense response to virus. Additionally, IFN-γ might play a crucial role in DHAV-1 CH60 infection. Four key chemokines, *CCL4* (FC = 372.7), *CCL19* (FC = 128.5), *CCL26* (FC = 18.9), and *CX3CL1* (FC = 20.5), participate in the cellular response to IFN-γ (Table S3 in Supplementary Material). Among the abovementioned biological processes, defense response to virus was the only common biological process during CH strain infection (Figure S4A in Supplementary Material). Furthermore, by analyzing GO enrichment of DEGs between the CH group and the CH60 group, we found that the identified biological processes almost completely overlapped with those identified by GO enrichment of DEGs between the mock group and the CH60 group (Figure 5B).

**Figure 6:**
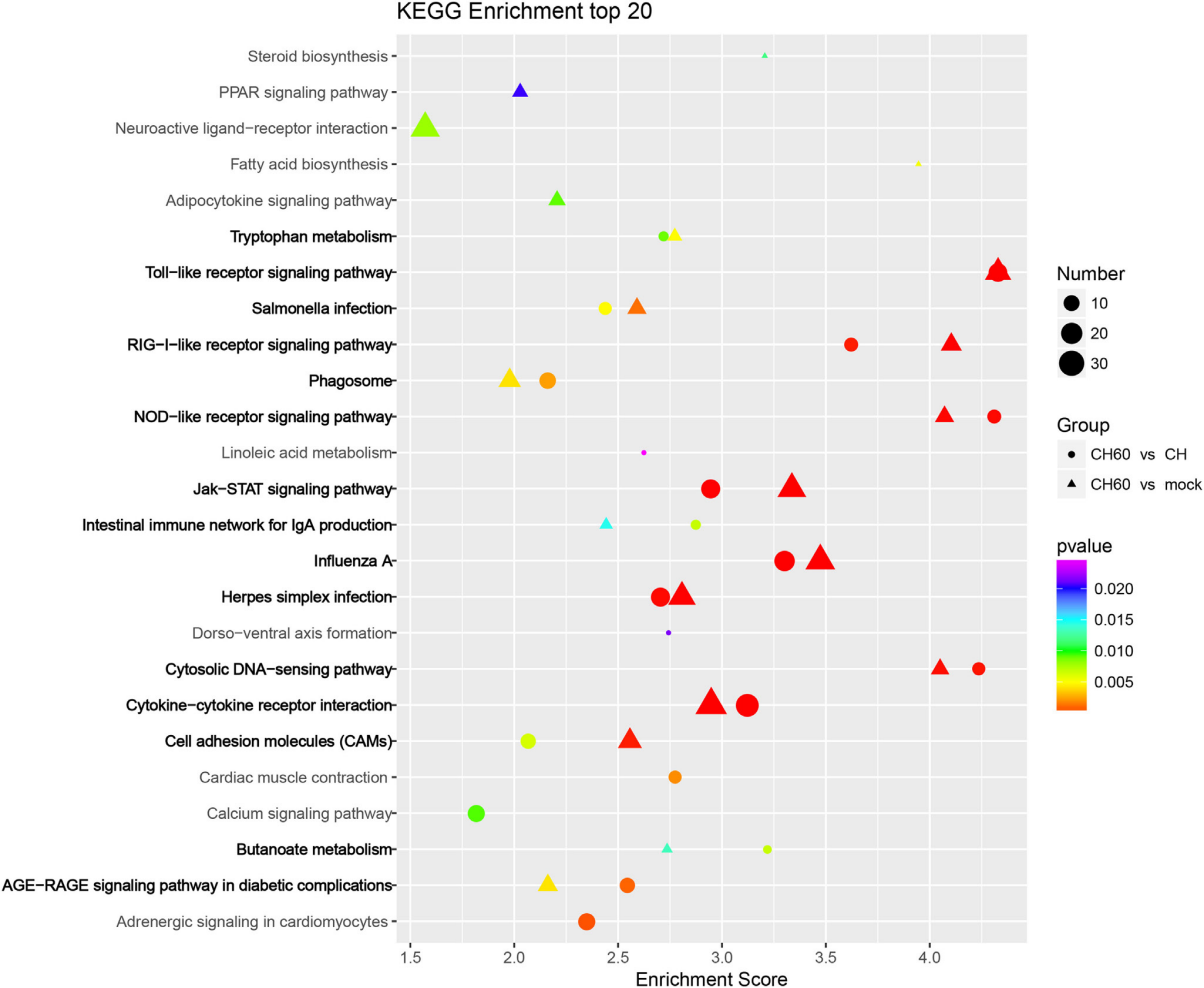
KEGG enrichment analysis of differentially expressed genes (DEGs) (*p*-value < 0.01 and fold change > 4). Circles and triangles represent KEGG enrichment analyses of the DEGs between the CH and CH60 groups and the DEGs between the mock and CH60 groups, respectively. The colors of the circles and triangles indicate *p*-value; the sizes indicate the number of genes assigned to the term.

Three pattern recognition receptor signaling pathways of the innate immune system were activated by CH60 infection, namely, the toll-like receptor signaling pathway (*TLR3, MYD88, IKK*ε, *IRF7*, and *MD-2*), the retinoic acid-inducible gene I (RIG-I)-like receptor signaling pathway (*MDA5, LGP2, TRIM25*, and *STING*), and the nucleotide oligomerization domain (NOD)-like receptor signaling pathway (*RIPK2, Caspase1*, and *TNFAIP3*); another key signaling pathway, the JAK–STAT signaling pathway (*SOCS1, SOCS3, STAT1, STAT2*, and *STAT4*), was also involved (Figure 6).

Duck hepatitis A virus type 1 CH60 infection led to abnormalities in multiple metabolic pathways in the liver (Figure 6). For example, in tryptophan metabolism (Figure S5A in Supplementary Material), the expression of tryptophan hydroxylase 1 (*TPH1*, FC = 61) and tryptophan 2,3-dioxygenase (*TDO2*, FC = 4.2) was upregulated, potentially affecting tryptophan biosynthesis after CH60 infection. In terms of fatty acid biosynthesis (Figure S5B in Supplementary Material), we found that expression of the key protein fatty acid synthase (*FASN*) was 10-fold higher after CH60 infection, potentially stimulating fatty acid synthesis. This finding was consistent with the observation of large numbers of fat particles by TEM. In addition, we were interested in a vacuolar ATPase, *ATP6V1G3* (FC = 873), in the phagosome membrane (Figure S5C in Supplementary Material), which played an important role in the evolution of the phagosome (38), consistent with observations of the phagosome by TEM.

### Confirmation of Differential Gene Expression and Methylation Status of SOCS3

**Figure 7:**
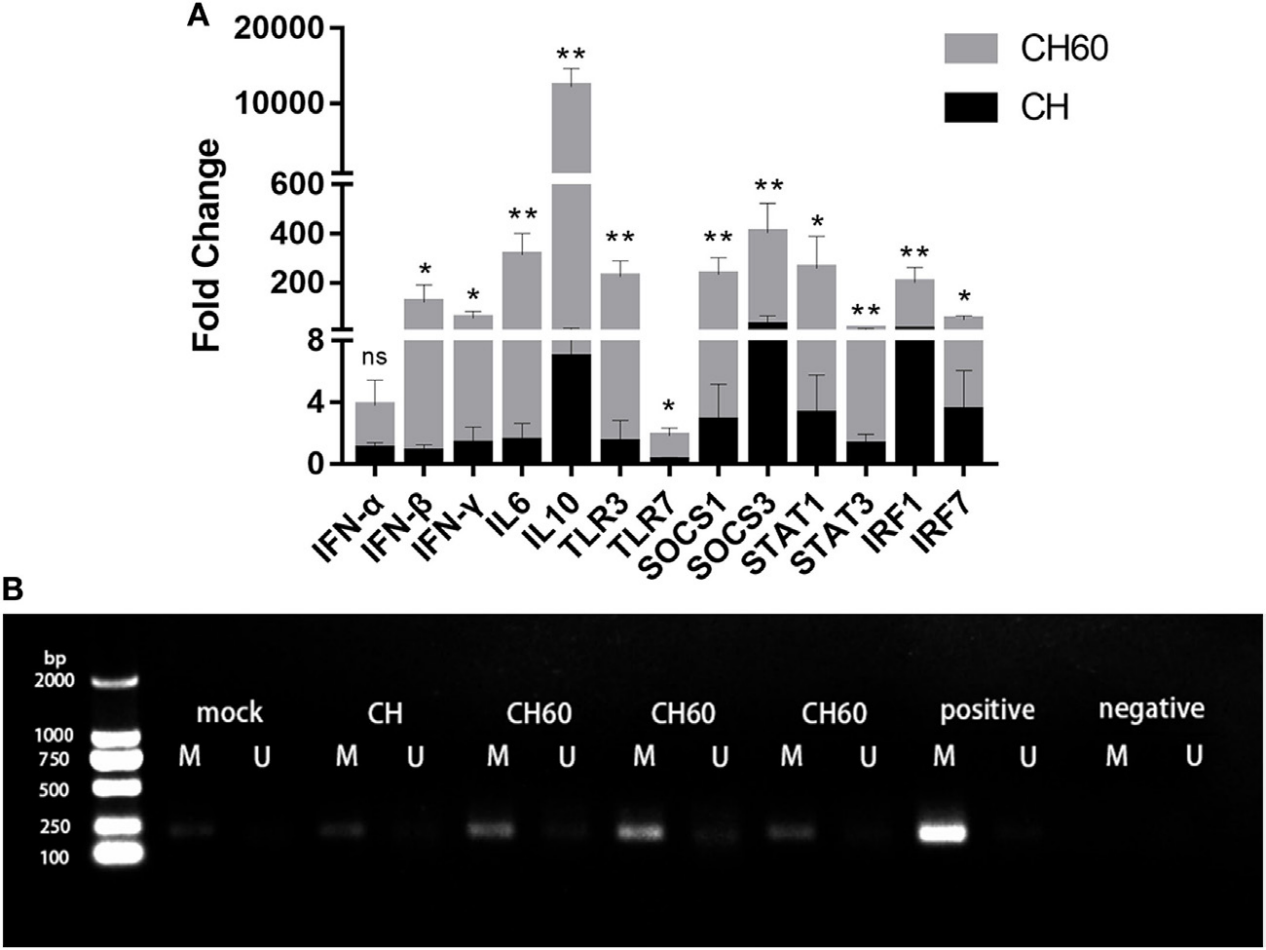
Confirmation of differentially expressed genes by RT-PCR and methylation status of *SOCS3*. **(A)** Gene expression levels were measured by the 2^−ΔΔCt^ method with relative quantification. Differences in the expression levels of the various genes between strains CH and CH60 were analyzed using Student’s *t*-test and were considered significant as follows: *p < 0.05; **p < 0.01; **(B)** Methylation status of the *SOCS3* gene in liver tissues from Duck hepatitis A virus type 1 (DHAV-1)-infected embryonated eggs. DNA from the liver tissues of embryonated eggs infected with DHAV-1 at 48 hpi was subjected to methylation-specific PCR. M, methylation-specific primers; U, non-methylation-specific primers.

We confirmed expression of 13 critical DEGs, namely, *SOCS-1, SOCS-3, STAT1, STAT3, IRF-1, IRF-7, IFN-*α, *IFN-*β, *IFN-*γ, *IL-6, IL-10, TLR3*, and *TLR7*, by qRT-PCR in the same samples. Compared to the CH group, gene expression was significantly higher in the CH60 group (Figure 7A). Importantly, the genes identified by qRT-PCR exhibited similar expression levels as those detected *via* transcriptomic analysis. Furthermore, we determined the methylation status of *SOCS3* in the livers of the mock, CH, and CH60 groups. All samples showed *SOCS3* hypermethylation (Figure 7B).

## Discussion

Herein, we describe the first global intrahepatic transcriptional profiles of embryonated eggs infected with the virulent DHAV-1 CH strain or the attenuated CH60 commercial vaccine strain and combined the results with microstructural and ultrastructural analyses to compare the effects of the two strains. Notably, the hepatic sinusoids were infiltrated by large numbers of red blood cells, which was accompanied by disordered hepatic cords and granular degeneration in the livers of CH60-infected embryonated eggs, consistent with liver injury in duck embryos infected with DHAV-1 (21). We also observed abundant lysosomes and secondary lysosomes in TEM images of the CH60 group (Figure 2). A similar hepatotropic virus, hepatitis A virus, utilized lysosomal organelles to facilitate nonlytic viral release, and final maturation was catalyzed by lysosomal proteases (39). Therefore, we hypothesized that a similar phenomenon occurred in the livers of DHAV-1-infected embryonated eggs. Moreover, the mitochondria in the livers of the CH60 group were slightly swollen where the ridge was fractured (Figure 2), which was consistent with mitochondrial injury in ducks infected with DHAV-1 (40). The mitochondrial apoptosis pathway is the main pathway of apoptosis, and damaged mitochondria are likely to affect apoptosis (41, 42). There was obvious apoptosis in the livers of CH60-infected embryonated eggs. Notably, several apoptotic cells clung to hepatocytes, and we speculate that these cells might be apoptotic hepatic stellate cells. The current study revealed that the liver can repair fibrosis by inducing the apoptosis of hepatic stellate cells (43, 44).

The fundamental role of the innate immune system is to detect invading viruses, induce inflammatory responses, produce cytokines, and chemokines to fight against viral infection, and most importantly, activate adaptive immunity (45, 46). Invading viruses are detected by pattern-recognition receptors (PRRs), which include toll-like receptors, RIG-I-like receptors, and NOD-like receptors. We found that the CH60 strain could be detected by multiple PRRs, especially TLR3 and TLR7. Once TLR3 and TLR7 sense viral RNA, downstream molecules (IRF3 and IRF7) are activated, resulting in induction of type I IFN, including IFN-α and IFN-β. This mechanism corresponds with our results (Figure 7). The inflammatory response is a double-edged sword that plays an important role in liver metabolism. A mild inflammatory response exerts consistent hepatoprotective effects. Conversely, excessive inflammation may cause liver damage (47). Previous studies have indicated that DHAV-1 induces excessive inflammatory responses and causes tissue damage in the liver (21, 48) and kidney (1). In our study, a pro-inflammatory mediator (*IL-6*) and an anti-inflammatory mediator (*IL-10*) were significantly upregulated during CH60 infection, combined with severe pathological damage. Inflammation is known to be driven by apoptosis in the liver (49). Apoptosis was visibly induced by the CH60 strain. Therefore, we hypothesized that CH60 infection induced apoptosis in the liver, that hepatocyte apoptosis drove inflammation in the liver, and that excessive inflammatory responses caused liver damage. To directly attack invading viruses, the innate immune system produces a mass of cytokines and chemokines. Based on GO enrichment analysis, genes expressing cytokines (*IFN-*β, *IFN-*γ, *OASL, IFIT5*) and chemokines (*CCL4, CCL19, CCL26*, and *CX3CL1*) were significantly upregulated and played an antiviral role during CH60 infection.

Notably, expression of *IL-10* was significantly upregulated in the livers of CH60-infected embryonated eggs. As an anti-inflammatory mediator, IL-10 suppresses inflammation *via* various mechanisms, including reducing HLA class II expression, decreasing T cell secretion of IL-2, and diminishing the production of IL-1, TNF-α, and IL-8 by activated monocytes/macrophages (50, 51). Furthermore, we found that expression of *IL-1, IL-2, TNF-*α, and *IL-8* was not significantly changed in CH60-infected livers (Table S1 in Supplementary Material); therefore, these cytokines may be suppressed by excessive expression of IL-10. Another key cytokine, IFN-γ, plays a vital role in CH60 infection. Unlike type I interferon, IFN-γ participates in innate and adaptive immunity by regulating the differentiation of natural T cells (52). According to studies investigating hepatitis E virus, high levels of TLR3 and a robust IFN-γ response are able to limit the disease, and hosts can recover uneventfully (53). Similar expression of *TLR3* and *IFN-*γ was observed in our studies; however, the liver did not recover uneventfully, and we speculate that the virus may evade the innate immune system through an unknown mechanism.

Interestingly, we observed that expression of *SOCS1* and *SOCS3* was significantly upregulated in the livers of CH60-infected embryonated eggs; hypermethylation of *SOCS3* was confirmed. These two molecules are involved in the cytokine-mediated signaling pathway according to GO enrichment. Notably, a similar phenomenon was observed in multiple hepatitis models (54–56). SOCS proteins were shown to be negative-feedback regulators of cytokine signaling mediated by the JAK–STAT signaling pathway, and cytokine signaling played an important role in the differentiation, maturation, proliferation, and apoptosis of various types of cells (57). The current study revealed that SOCS1 reduces induction of the IFN signaling pathway in chicken cells and can potentiate virus replication (58). A similar study revealed that *SOCS1* downregulation by miRNA 155 enhances type I IFN expression and suppresses virus replication (59). In our study, we hypothesized that the CH60 strain may utilize SOCS1 to suppress expression of IFN-α, ultimately facilitating replication; however, further research is needed to verify this hypothesis. The JAK–STAT signaling pathway is a central communication node for the immune system (60). KEGG analysis indicated that the JAK–STAT signaling pathway played a critical role in the livers of CH60-infected embryonated eggs. Expression of *STAT1* and *STAT3*, two key genes in the JAK–STAT signaling pathway, was markedly upregulated. STAT3 plays an important role in liver inflammation and cancer. The pro-inflammatory mediator IL-6 exerts many of its functions *via* activation of STAT3 (61). A study investigating HCV revealed that HCV repressed the cellular antiviral response by upregulating *STAT3* (62). However, interactions among SOCS, IFN, and STAT during DHAV-1 infection are not fully understood, and further research is needed.

In summary, our study is the first to utilize the RNA-Seq platform for an in-depth exploration of virus–host interactions, and we identified a number of genes that were dysregulated during CH60 infection and jointly analyzed these genes with pathological lesions and apoptosis. Excessive inflammatory and innate immune responses induced by the CH60 strain caused severe liver damage. The alternative animal model described in this study will be used for further molecular exploration of viral hepatitis and to elucidate the pathogenesis of DHAV-1.

## Ethics Statement

The study was approved by the Committee of Experiment Operational Guidelines and Animal Welfare of Sichuan Agricultural University (the approved permit number is XF2014-18). Experiments were conducted in accordance with approved guidelines.

## Author Contributions

JX carried out the experiments; AC, MW, JX, and XO conceived and supervised the study; JX, QZ, and MW drafted the manuscript; JX, YM, XZ, and ML recorded gross lesions and analyzed histopathology; JX, DZ, SC, RJ, and QY analyzed the transcriptomic data and confirmed differential gene expression; and YW, SZ, YL, YY, XC, and LZ revised the manuscript. All authors reviewed the manuscript.

## Conflict of Interest Statement

The authors declare that the research was conducted in the absence of any commercial or financial relationships that could be construed as a potential conflict of interest.

## References

[1] Ou X Mao S Jiang Y Zhang S Ke C Ma G Viral-host interaction in kidney reveals strategies to escape host immunity and persistently shed virus to the urine. Oncotarget (2017) 8(5):7336–49. 10.18632/oncotarget.14227 28038465 PMC5352325

[2] Qianda C Anchun C Mingshu W Characteristics and function of 3D gene and its encoding protein in picornavirus. Rev Med Microbiol (2012) 23(2):18–22. 10.1097/MRM.0b013e328352afee

[3] Cao J Ou X Zhu D Ma G Cheng A Wang M The 2A2 protein of duck hepatitis A virus type 1 induces apoptosis in primary cell culture. Virus Genes (2016) 52(6):780–8. 10.1007/s11262-016-1364-4 27314270

[4] Cao QD Cheng AC Wang MS Bioinformatic analysis of the 3D polyprotein from duck hepatitis A virus strain H isolated in China. Adv Mater Res (2013) 647:396–402. 10.4028/www.scientific.net/AMR.647.403

[5] Sun D Chen S Cheng A Wang M. Roles of the picornaviral 3C proteinase in the viral life cycle and host cells. Viruses (2016) 8(3):82. 10.3390/v8030082 26999188 PMC4810272

[6] Wen X Cheng A Wang M Jia R Zhu D Chen S Recent advances from studies on the role of structural proteins in enterovirus infection. Future Microbiol (2015) 10(9):1529–42. 10.2217/fmb.15.62 26343779

[7] Yang X Cheng A Wang M Jia R Sun K Pan K Structures and corresponding functions of five types of picornaviral 2A proteins. Front Microbiol (2017) 8:1373. 10.3389/fmicb.2017.01373 28785248 PMC5519566

[8] Sun D Wang M Wen X Cheng A Jia R Sun K Cleavage of poly(A)-binding protein by duck hepatitis A virus 3C protease. Sci Rep (2017) 7(1):16261. 10.1038/s41598-017-16484-1 29176600 PMC5701138

[9] Levine P Hofstad M Report of the poultry diagnosis laboratory at Ithaca. Report of the New York State Veterinary College at Cornell University for the Year 1944–1945; Ithaca. Geneva, New York: W. F. Humphrey Press Inc. (1946). p. 54–60.

[10] Wen X Zhu D Cheng A Wang M Chen S Jia R Molecular epidemiology of duck hepatitis a virus types 1 and 3 in China, 2010-2015. Transbound Emerg Dis (2018) 65(1):10–5. 10.1111/tbed.12741 29076646

[11] Kamomae M Kameyama M Ishii J Nabe M Ogura Y Iseki H An outbreak of duck hepatitis A virus type 1 infection in Japan. J Vet Med Sci (2017) 79(5):917–20. 10.1292/jvms.16-0646 28413174 PMC5447982

[12] Tsai H-J Woolcock P Viral infections of waterfow. In: Swayne DE Glisson JR McDougald LR Nolan LK Suarez DL Nair VL editors. Diseases of Poultry. Vol. 13 Oxford, UK: John Wiley & Sons, Inc. (2013). p. 417–63.

[13] Yugo DM Hauck R Shivaprasad HL Meng XJ. Hepatitis virus infections in poultry. Avian Dis (2016) 60(3):576–88. 10.1637/11229-070515-Review.1 27610716

[14] Song C Yu S Duan Y Hu Y Qiu X Tan L Effect of age on the pathogenesis of DHV-1 in Pekin ducks and on the innate immune responses of ducks to infection. Arch Virol (2014) 159(5):905–14. 10.1007/s00705-013-1900-7 24162826

[15] Mao S Ou X Zhu D Chen S Ma G Wang M Development and evaluation of indirect ELISAs for the detection of IgG, IgM and IgA1 against duck hepatitis A virus 1. J Virol Methods (2016) 237:79–85. 10.1016/j.jviromet.2016.08.019 27577105

[16] Hu Q Zhu D Ma G Cheng A Wang M Chen S A one-step duplex rRT-PCR assay for the simultaneous detection of duck hepatitis A virus genotypes 1 and 3. J Virol Methods (2016) 236:207–14. 10.1016/j.jviromet.2016.07.011 27435338

[17] Wen XJ Cheng AC Wang MS Jia RY Zhu DK Chen S. Detection, differentiation, and VP1 sequencing of duck hepatitis A virus type 1 and type 3 by a 1-step duplex reverse-transcription PCR assay. Poult Sci (2014) 93(9):2184. 10.3382/ps.2014-04024 25012848

[18] Shen Y Cheng A Wang M Chen S Jia R Zhu D Development of an indirect ELISA method based on the VP3 protein of duck hepatitis A virus type 1 (DHAV-1) for dual detection of DHAV-1 and DHAV-3 antibodies. J Virol Methods (2015) 225:30–4. 10.1016/j.jviromet.2015.08.016 26341062

[19] Anchun C Mingshu W Hongyi X Dekang Z Xinran L Haijuen C Development and application of a reverse transcriptase polymerase chain reaction to detect Chinese isolates of duck hepatitis virus type 1. J Microbiol Methods (2009) 77(3):332–6. 10.1016/j.mimet.2009.02.002 19475729

[20] Yang M Cheng A Wang M Xing H. Development and application of a one-step real-time Taqman RT-PCR assay for detection of Duck hepatitis virus type1. J Virol Methods (2008) 153(1):55–60. 10.1016/j.jviromet.2008.06.012 18611411

[21] Ou X Mao S Cao J Ma Y Ma G Cheng A The neglected avian hepatotropic virus induces acute and chronic hepatitis in ducks: an alternative model for hepatology. Oncotarget (2017) 8(47):81838–51. 10.18632/oncotarget.19003 29137226 PMC5669852

[22] Ou X Mao S Cao J Cheng A Wang M Zhu D Comparative analysis of virus-host interactions caused by a virulent and an attenuated duck hepatitis A virus genotype 1. PLoS One (2017) 12(6):e0178993. 10.1371/journal.pone.0178993 28614378 PMC5470708

[23] Mao S Wang M Ou X Sun D Cheng A Zhu D Virologic and immunologic characteristics in mature ducks with acute duck hepatitis A virus 1 infection. Front Immunol (2017) 8:1574. 10.3389/fimmu.2017.01574 29201029 PMC5696325

[24] Song C Liao Y Gao W Yu S Sun Y Qiu X Virulent and attenuated strains of duck hepatitis A virus elicit discordant innate immune responses in vivo. J Gen Virol (2014) 95(12):2716–26. 10.1099/vir.0.070011-0 25217614

[25] Kain KH Miller JW Jones-Paris CR Thomason RT Lewis JD Bader DM The chick embryo as an expanding experimental model for cancer and cardiovascular research. Dev Dyn (2014) 243(2):216–28. 10.1002/dvdy.24093 24357262 PMC4164046

[26] Strojny B Grodzik M Sawosz E Winnicka A Kurantowicz N Jaworski S Diamond nanoparticles modify curcumin activity: in vitro studies on cancer and normal cells and in ovo studies on chicken embryo model. PLoS One (2016) 11(10):e0164637. 10.1371/journal.pone.0164637 27736939 PMC5063465

[27] Beedie SL Rore HM Barnett S Chau CH Luo W Greig NH In vivo screening and discovery of novel candidate thalidomide analogs in the zebrafish embryo and chicken embryo model systems. Oncotarget (2016) 7(22):33237–45. 10.18632/oncotarget.8909 27120781 PMC5078090

[28] Vancamp P Darras VM. Dissecting the role of regulators of thyroid hormone availability in early brain development: merits and potential of the chicken embryo model. Mol Cell Endocrinol (2017) 459:71–8. 10.1016/j.mce.2017.01.045 28153797

[29] Peng M Li S He Q Zhao J Li L Ma H. Proteomics reveals changes in hepatic proteins during chicken embryonic development: an alternative model to study human obesity. BMC Genomics (2018) 19(1):29. 10.1186/s12864-017-4427-6 29310583 PMC5759888

[30] Cheng A Liao D Xie J Chen X Studies on duck viral hepatitis – pathogen isolation, identification and cultivation of attenuated viruses [J]. China J Vet Med (1993) 19(1):3–4.

[31] Cardiff RD Miller CH Munn RJ. Manual hematoxylin and eosin staining of mouse tissue sections. Cold Spring Harb Protoc (2014) 2014(6):655–8. 10.1101/pdb.prot073411 24890205

[32] Livak KJ Schmittgen TD. Analysis of relative gene expression data using real-time quantitative PCR and the 2(-Delta Delta C(T)) Method. Methods (2001) 25(4):402–8. 10.1006/meth.2001.1262 11846609

[33] Kim D Langmead B Salzberg SL. HISAT: a fast spliced aligner with low memory requirements. Nat Methods (2015) 12(4):357–60. 10.1038/nmeth.3317 25751142 PMC4655817

[34] Langmead B Salzberg SL. Fast gapped-read alignment with Bowtie 2. Nat Methods (2012) 9(4):357–9. 10.1038/nmeth.1923 22388286 PMC3322381

[35] Roberts A Pachter L. Streaming fragment assignment for real-time analysis of sequencing experiments. Nat Methods (2013) 10(1):71–3. 10.1038/nmeth.2251 23160280 PMC3880119

[36] Anders S Huber W Differential expression of RNA-Seq data at the gene level–the DESeq package. EMBL. Heidelberg, Germany (2012). Available from: http://gga01.med.wayne.edu/online_help/help_regionminer/DESeq.pdf

[37] Kanehisa M Araki M Goto S Hattori M Hirakawa M Itoh M KEGG for linking genomes to life and the environment. Nucleic Acids Res (2008)36(Database issue):480–4. 10.1093/nar/gkm882 18077471 PMC2238879

[38] Fairn GD Grinstein S. How nascent phagosomes mature to become phagolysosomes. Trends Immunol (2012) 33(8):397–405. 10.1016/j.it.2012.03.003 22560866

[39] Seggewiß N Paulmann D Dotzauer A. Lysosomes serve as a platform for hepatitis A virus particle maturation and nonlytic release. Arch Virol (2016) 161(1):43–52. 10.1007/s00705-015-2634-5 26467925

[40] Du H Yang J Bai J Ming K Shi J Yao F A flavone-polysaccharide based prescription attenuates the mitochondrial dysfunction induced by duck hepatitis A virus type 1. PLoS One (2017) 12(4):e0175495. 10.1371/journal.pone.0175495 28394931 PMC5386289

[41] Malhi H Gores GJ Lemasters JJ. Apoptosis and necrosis in the liver: a tale of two deaths? Hepatology (2006) 43(2 Suppl 1):S31. 10.1002/hep.21062 16447272

[42] You Y Cheng AC Wang MS Jia RY Sun KF Yang Q The suppression of apoptosis by α-herpesvirus. Cell Death Dis (2017) 8(4):e2749. 10.1038/cddis.2017.139 28406478 PMC5477576

[43] Modol T Brice N Ruiz de Galarreta M Garcia Garzon A Iraburu MJ Martinez-Irujo JJ Fibronectin peptides as potential regulators of hepatic fibrosis through apoptosis of hepatic stellate cells. J Cell Physiol (2015) 230(3):546–53. 10.1002/jcp.24714 24976518

[44] Issa R Williams E Trim N Kendall T Arthur MJ Reichen J Apoptosis of hepatic stellate cells: involvement in resolution of biliary fibrosis and regulation by soluble growth factors. Gut (2001) 48(4):548–57. 10.1136/gut.48.4.548 11247901 PMC1728234

[45] Szabo G Mandrekar P Dolganiuc A. Innate immune response and hepatic inflammation. Semin Liver Dis (2007) 27(4):339–50. 10.1055/s-2007-991511 17979071

[46] Takeuchi O Akira S. Innate immunity to virus infection. Immunol Rev (2009) 227(1):75–86. 10.1111/j.1600-065X.2008.00737.x 19120477 PMC5489343

[47] Brenner C Galluzzi L Kepp O Kroemer G. Decoding cell death signals in liver inflammation. J Hepatol (2013) 59(3):583–94. 10.1016/j.jhep.2013.03.033 23567086

[48] Xie J Wang M Cheng A Zhao XX Liu M Zhu D Cytokine storms are primarily responsible for the rapid death of ducklings infected with duck hepatitis A virus type 1. Sci Rep (2018) 8(1):6596. 10.1038/s41598-018-24729-w 29700351 PMC5920089

[49] Faouzi S Burckhardt BE Hanson JC Campe CB Schrum LW Rippe RA Anti-Fas induces hepatic chemokines and promotes inflammation by an NF-kappa B-independent, caspase-3-dependent pathway. J Biol Chem (2001) 276(52):49077–82. 10.1074/jbc.M109791200 11602613

[50] Tilg H Kaser A Moschen AR. How to modulate inflammatory cytokines in liver diseases. Liver Int (2006) 26(9):1029–39. 10.1111/j.1478-3231.2006.01339.x 17032402

[51] Mosmann TR Properties and functions of interleukin-10. Adv Immunol (1994) 56:1–26. 10.1016/S0065-2776(08)60449-6 8073945

[52] Schoenborn JR Wilson CB. Regulation of interferon-gamma during innate and adaptive immune responses. Adv Immunol (2007) 96:41–101. 10.1016/S0065-2776(07)96002-2 17981204

[53] Majumdar M Ratho RK Chawla Y Singh MP. Role of TLR gene expression and cytokine profiling in the immunopathogenesis of viral hepatitis E. J Clin Virol (2015) 73:8–13. 10.1016/j.jcv.2015.09.011 26512422

[54] Fletcher SP Chin DJ Ji Y Iniguez AL Taillon B Swinney DC Transcriptomic analysis of the woodchuck model of chronic hepatitis B. Hepatology (2012) 56(3):820–30. 10.1002/hep.25730 22431061 PMC3401284

[55] Um TH Kim H Oh BK Kim MS Kim KS Jung G Aberrant CpG island hypermethylation in dysplastic nodules and early HCC of hepatitis B virus-related human multistep hepatocarcinogenesis. J Hepatol (2011) 54(5):939–47. 10.1016/j.jhep.2010.08.021 21145824

[56] Zhao LJ He SF Wang W Ren H Qi ZT. Interferon alpha antagonizes STAT3 and SOCS3 signaling triggered by hepatitis C virus. Cytokine (2016) 80:48–55. 10.1016/j.cyto.2015.08.264 26945996

[57] Inagaki-Ohara K Kondo T Ito M Yoshimura A. SOCS, inflammation, and cancer. JAKSTAT (2013) 2(3):e24053. 10.4161/jkst.24053 24069550 PMC3772102

[58] Giotis ES Ross CS Robey RC Nohturfft A Goodbourn S Skinner MA. Constitutively elevated levels of SOCS1 suppress innate responses in DF-1 immortalised chicken fibroblast cells. Sci Rep (2017) 7(1):17485. 10.1038/s41598-017-17730-2 29235573 PMC5727488

[59] Wang B Fu M Liu Y Wang Y Li X Cao H gga-miR-155 enhances type i interferon expression and suppresses infectious burse disease virus replication via targeting SOCS1 and TANK. Front Cell Infect Microbiol (2018) 8:55. 10.3389/fcimb.2018.00055 29564226 PMC5845882

[60] Villarino A Kanno Y O’Shea J. Mechanisms and consequences of Jak-STAT signaling in the immune system. Nat Immunol (2017) 18(4):374–84. 10.1038/ni.3691 28323260 PMC11565648

[61] He G Karin M NF-kappaB and STAT3 – key players in liver inflammation and cancer. Cell Res (2011) 21(1):159–68. 10.1038/cr.2010.183 21187858 PMC3193410

[62] Xiong Y Zhang C Yuan J Zhu Y Tan Z Kuang X Hepatitis C virus represses the cellular antiviral response by upregulating the expression of signal transducer and activator of transcription 3 through sponging microRNA122. Mol Med Rep (2015) 11(3):1733–7. 10.3892/mmr.2014.2897 25377467 PMC4270330

