# Supplementary material for: Transcriptomic Characterization of a Chicken Embryo Model Infected With Duck Hepatitis A Virus Type 1
Source: Front Immunol. 2018 Aug 24;9:1845. doi: 10.3389/fimmu.2018.01845 (PMC6117380; doi:10.3389/fimmu.2018.01845)
Supplement: Supplementary file 3 [file presentation_1.PDF]

Supplementary Material

Transcriptomic characterization of a chicken embryo model infected with duck hepatitis A virus type 1

Jinyan Xie[1,2¶], Qiurui Zeng[4¶],Mingshu Wang[1,2,3¶], Xumin Ou[1,2¶], Yunchao Ma[1,2], Anchun Cheng[1,2,3*], Xin-Xin Zhao[1,2,3], Mafeng Liu[1,2,3], Dekang Zhu[2,3], Shun Chen[1,2,3], Renyong Jia[1,2,3], Qiao Yang[1,2,3], Ying Wu[1,2,3], Shaqiu Zhang[1,2,3],Yunya Liu[1,2,3], Yanling Yu[1,2,3], Ling Zhang[1,2,3], Xiaoyue Chen[1,2,3]

Corresponding authors: Anchun Cheng, chenganchun@vip.163.com

Supplementary Figures and Tables

A

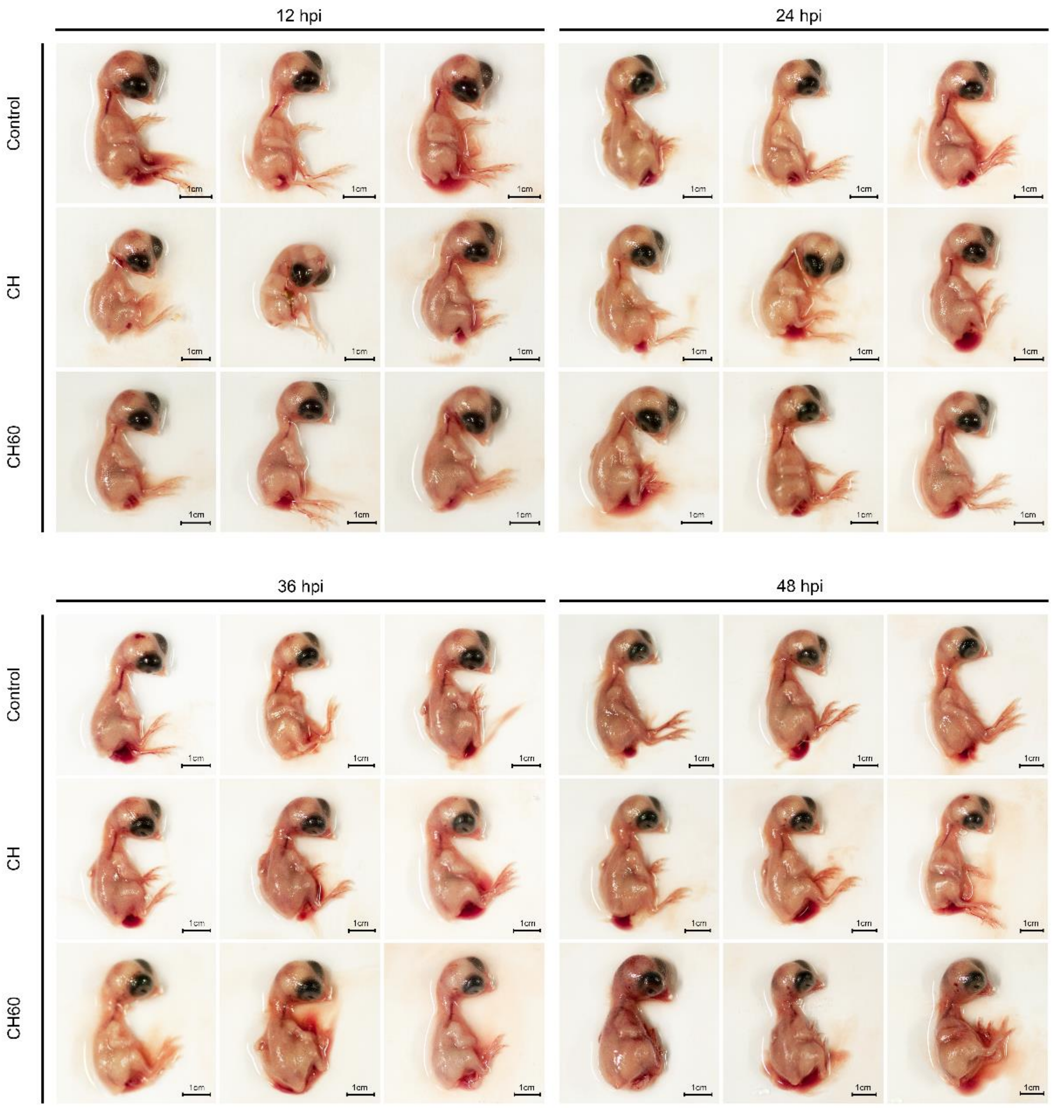

B

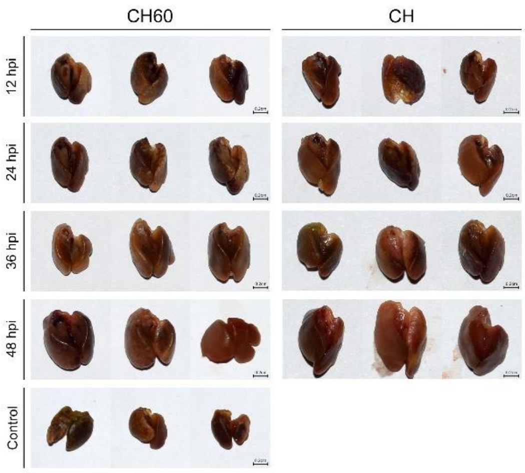

Supplementary Figure 1. Gross lesions and histopathological lesions in the CH- or CH60-infected livers of all chicken embryos. The chicken embryos were infected with the CH or CH60 strain (A), and then, the livers were collected and soaked in 4% paraformaldehyde solution at 12, 24, 36 and 48 hpi (B).

A

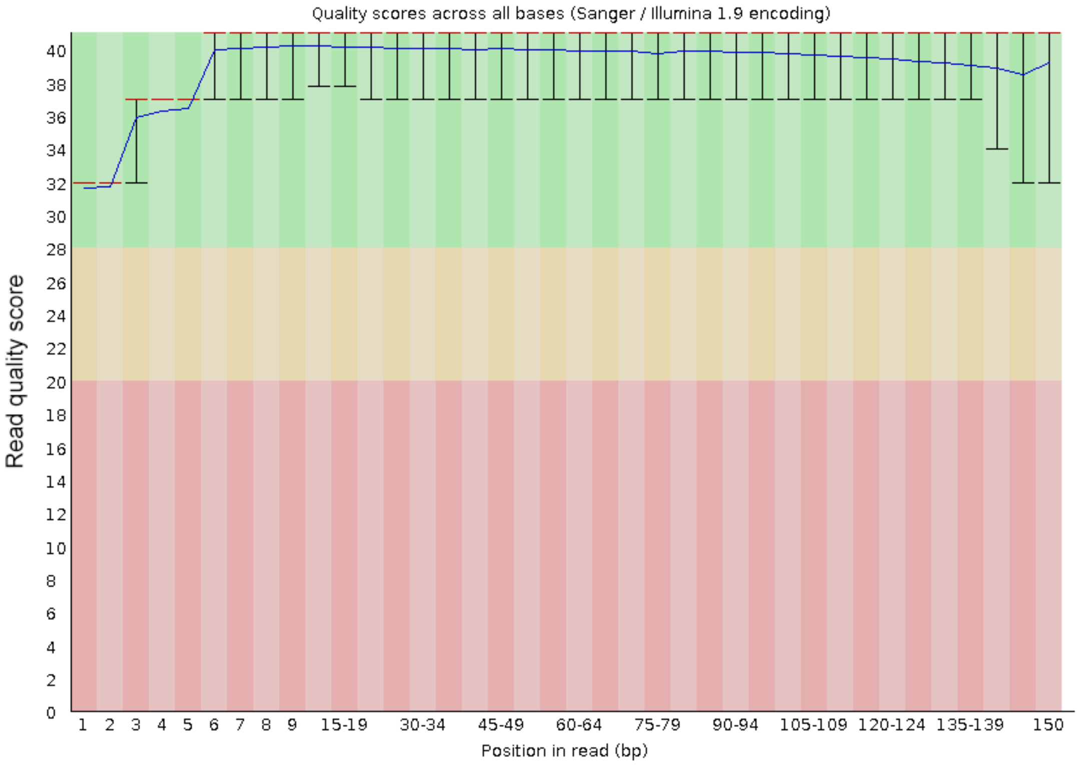
Quality scores across all bases (Sanger / Illumina 1.9 encoding)
Read quality score
0 2 4 6 8 10 12 14 16 18 20 22 24 26 28 30 32 34 36 38 40
1 2 3 4 5 6 7 8 9 15-19 30-34 45-49 60-64 75-79 90-94 105-109 120-124 135-139 150
Position in read (bp)

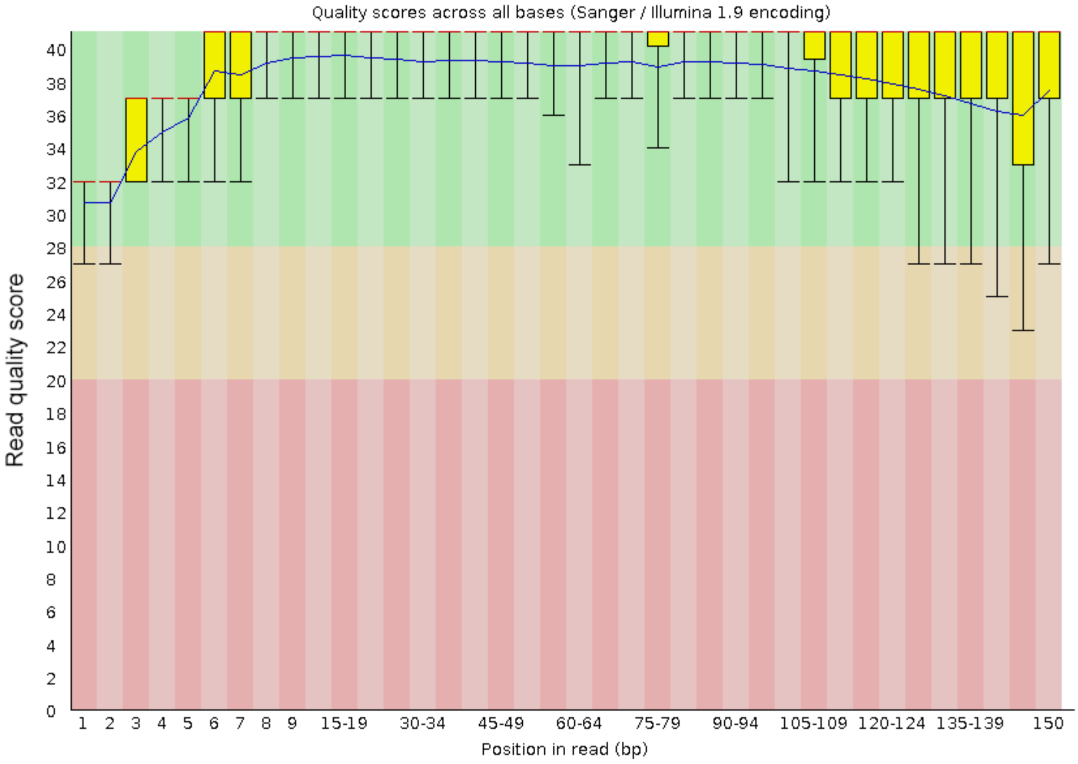
Quality scores across all bases (Sanger / Illumina 1.9 encoding)
Read quality score
0 2 4 6 8 10 12 14 16 18 20 22 24 26 28 30 32 34 36 38 40
1 2 3 4 5 6 7 8 9 15-19 30-34 45-49 60-64 75-79 90-94 105-109 120-124 135-139 150
Position in read (bp)

B

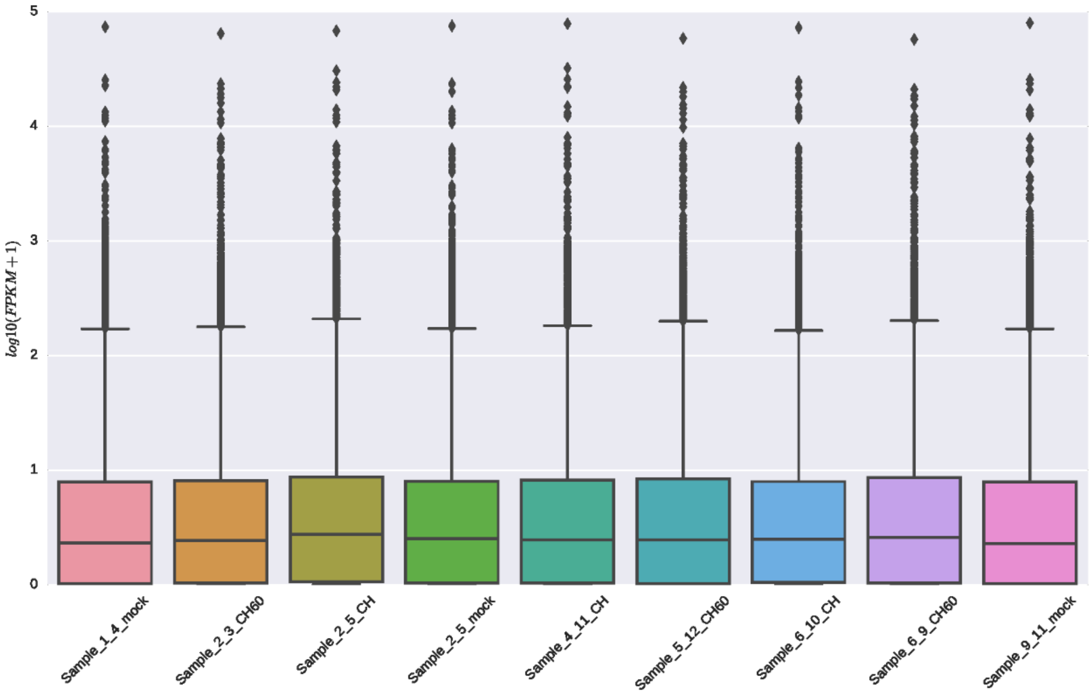

log10(FPKM+1)
5
4
3
2
1
0
Sample_1_4_mock
Sample_2_3_CH60
Sample_2_5_CH
Sample_2_5_mock
Sample_4_11_CH
Sample_5_12_CH60
Sample_6_10_CH
Sample_6_9_CH60
Sample_9_11_mock

C

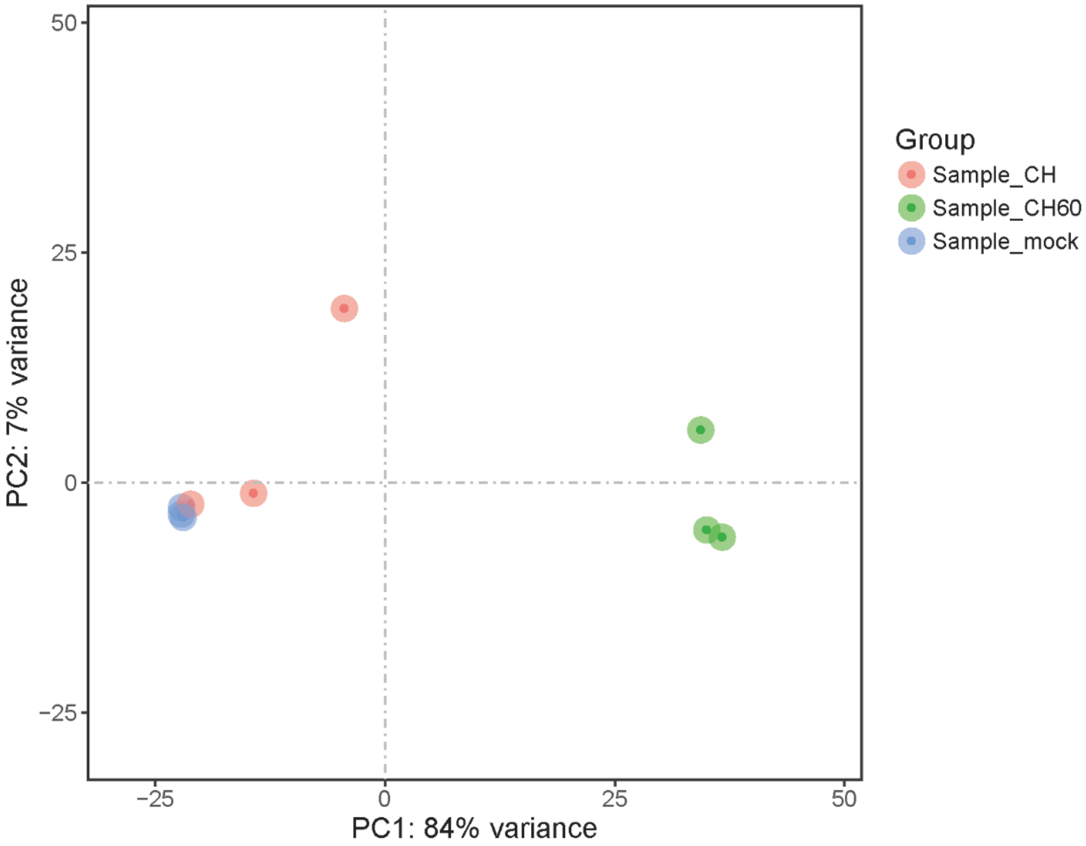

PC2: 7% variance
50
25
0
−25
−25
0
25
50
PC1: 84% variance
Group
Sample_CH
Sample_CH60
Sample_mock

D

**Sample**	**mock**	**CH**	**CH60**
Total reads; means of 3 biological replicates. (SD)	48623617(523872)	46896623(2145679)	48234173(862819)

Supplementary Figure 2. Quality control analyses and total reads data. (A) A raw data quality analysis across all bases. top graph for forward reads, the bottom graph reverse reads. The analysis was determined using the FastQC program (http://www.bioinformatics.babraham.ac.uk/projects/fastqc/) (v 0.11.5). The vast majority of reads were of high quality (green zone). The figure was representative of forward and reverse reads for all 9 sequencing samples. (B) Box-whisker Plot for all 9 sequencing samples. The abscissa represents the experimental conditions, and the ordinate represents the logarithm of FPKM+1. (C) PCA plot. PCA shows the relationship between samples from different dimensions. (D) total reads of three groups.

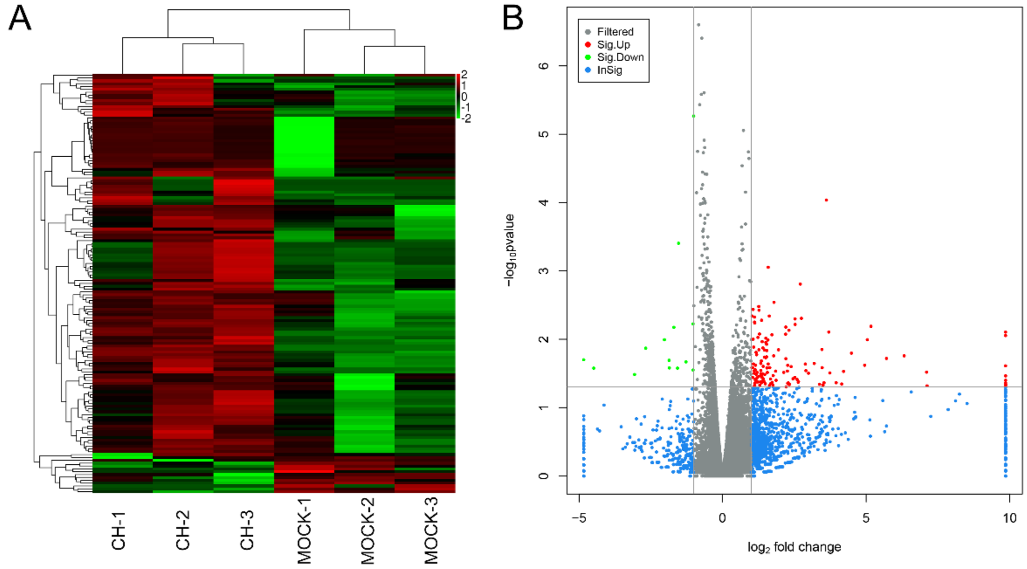

Supplementary Figure 3. Analysis of DEGs of the mock and CH groups (p-value<0.05 and fold change>2). A heatmap was used to classify the gene expression patterns, and a volcano plot displayed the number of DEGs. (A) X-axis represents the experimental conditions. (B) Volcano plot of DEGs between the CH and mock groups. Y-axis indicates the negative logarithm of the p-value; X-axis indicates the base 2 logarithm of fold change.

A

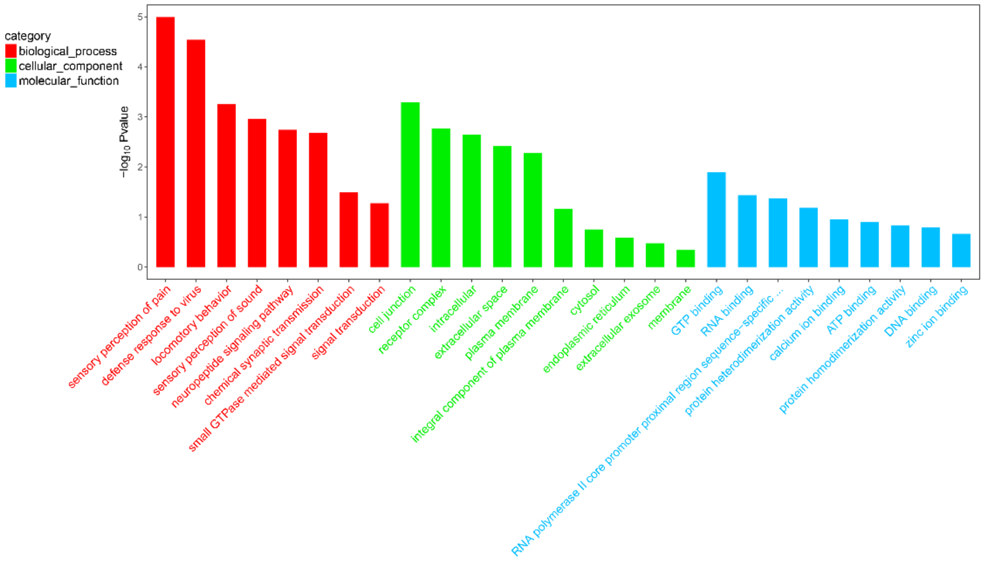

B

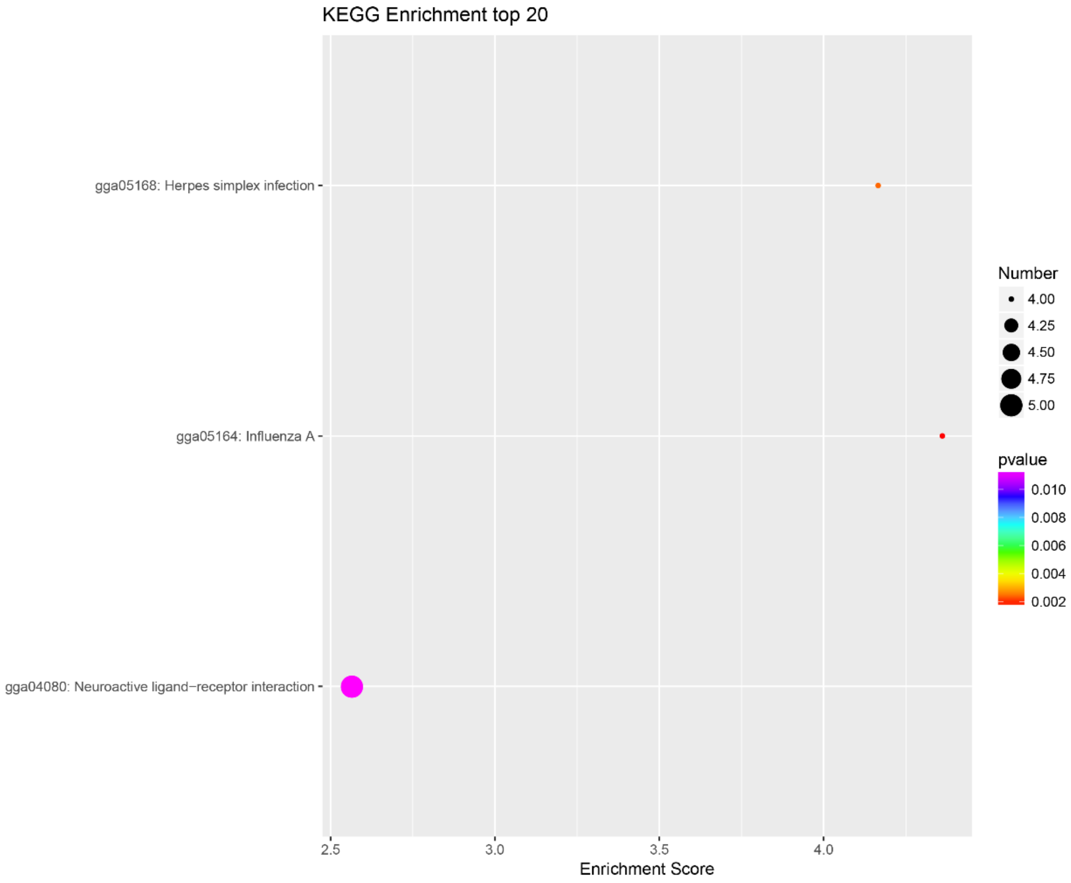

Supplementary Figure 4. GO enrichment (A) and KEGG enrichment (B) analysis of DEGs between the mock group and CH group (p-value<0.05 and fold change>2). (A) The categories are assigned to the x-axis, and the negative logarithm of the adjusted p-value is assigned to the y-axis. (B) Circles represent KEGG enrichment analyses of the

DEGs between the mock and CH groups. The colors of the circles indicate p-value; the sizes indicate the number of genes assigned to the term.

A tryptophan metabolism

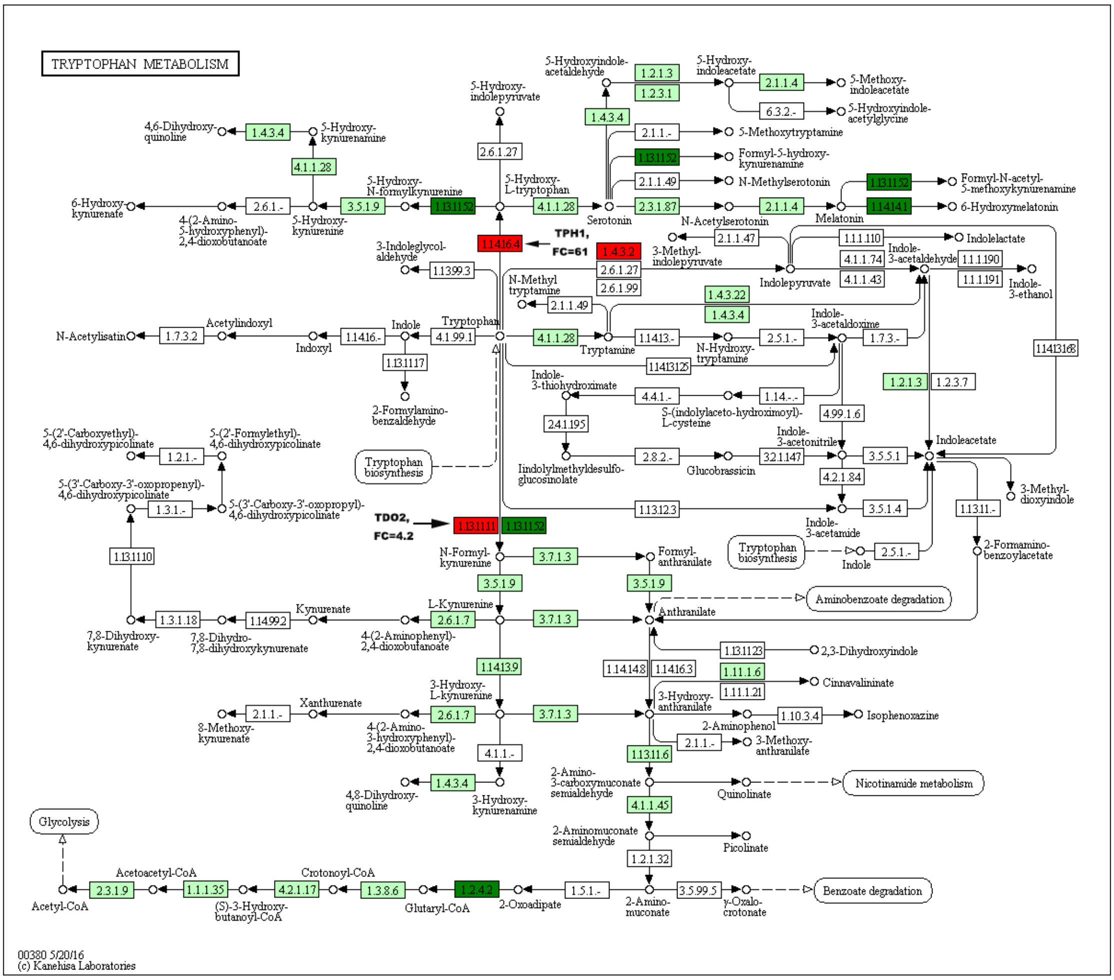

B fatty acid biosynthesis

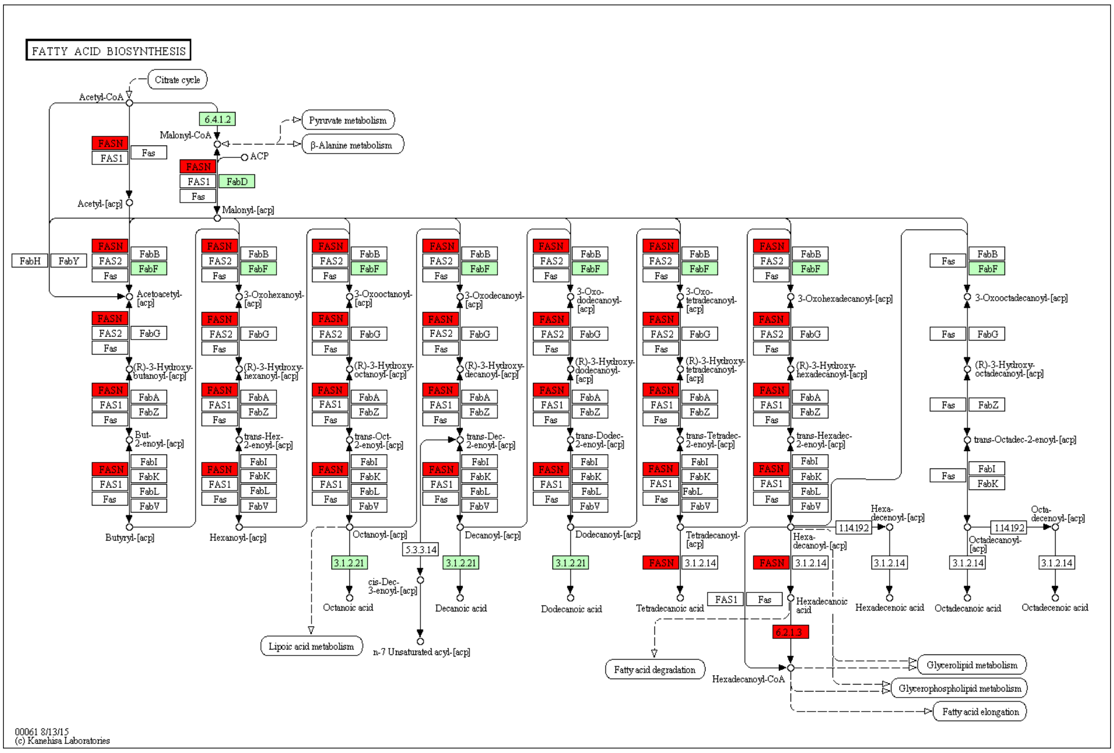

C phagosome

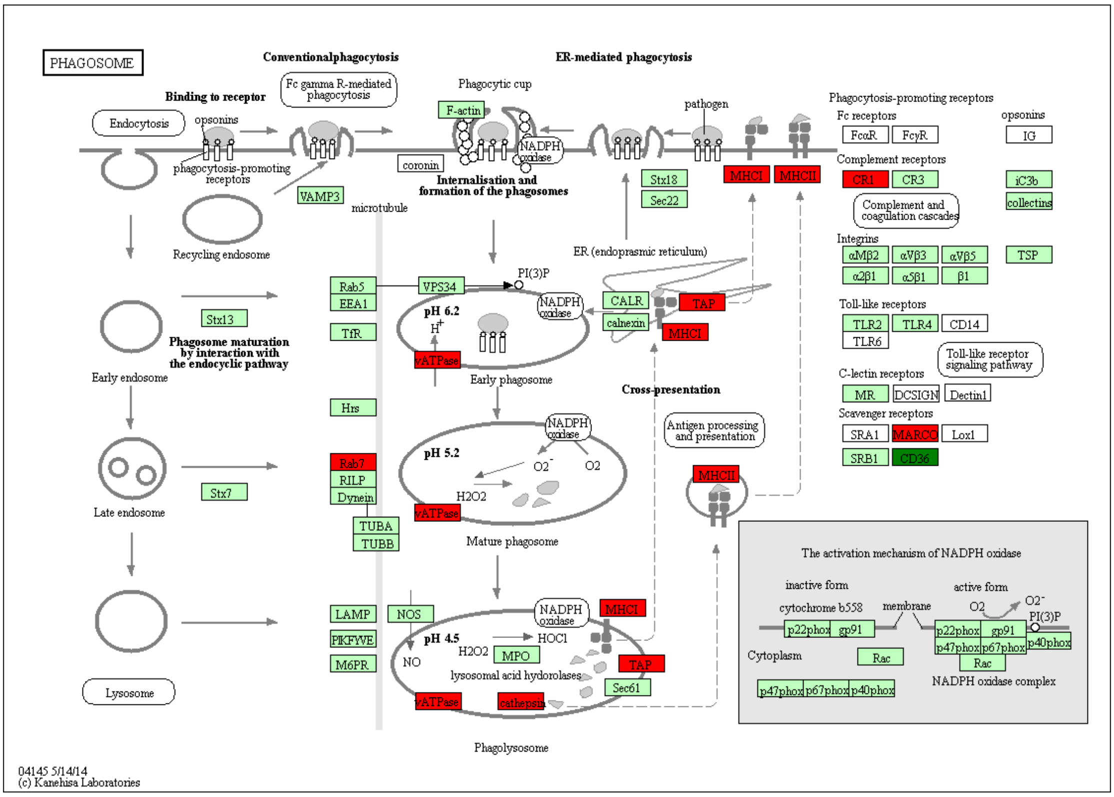

Supplementary Figure 5. The metabolic pathway in the process of CH60 infection. The red represented up-regulated and green represented down-regulated. (A) was the tryptophan metabolism. (B) was the fatty acid biosynthesis. (C) was the pathway of

phagosome

Supplementary Table 1. Sequences of primers used for gene expression profiling.

Gene	Forward (5′-3′)	Reverse (5′-3′)
IFN-α	TCGCAACCTTCACCTCACC	CGCAGGCGCTGTAATCGT
IFN-β	TCCAGCTCCTTCAGAATACG	TGCGGTCAATCCAGTGTT
IFN-γ	TCATACTGAGCCAGATTGT	AAGTCGTTCATCGGGAGC
TLR3	TGGCTAAACGACACTCAA	AGCTATTCTCCACCCTTC
TLR7	GACCCTGACTATTAACCAT	CGTAAAGTAGCAGGAAGAC
IL-6	GAAATCCCTCCTCGCCAATC	CCCTCACGGTCTTCTCCATAAA
IL-10	CAATCCAGGGACGATGAAC	GCAGGTGAAGAAGCGGTGA
SOCS1	GCACGCACTTCCGAACCT	ACACTGATGGCAAAGAAACAA
SOCS3	CGGCACTTCTTCACCCTCAG	CAGCTTCAGCACGCAGTCG
STAT1	GTAAAGAGGGAGCAATCA	TATCAGGGAAAGTAACAGC
STAT3	AAGCGTGGTCTCAGCATT	TGATTTGACAGCCCGAGTAG
IRF1	GGATGCTCCCACCTCTG	GTGCTGGTTAGTCGTTCTG
IRF7	AAAGCCCAAGGAGTCCAAGC	GCTGACGTTGCCACTGTTGA
GAPDH	TGAAAGTCGGAGTCAACGG	GGTCACGCTCCTGGAAGAT
